# Electronic Patient-Generated Health Data to Facilitate Disease Prevention and Health Promotion: Scoping Review

**DOI:** 10.2196/13320

**Published:** 2019-10-14

**Authors:** Vasileios Nittas, Penny Lun, Frederic Ehrler, Milo Alan Puhan, Margot Mütsch

**Affiliations:** 1 Epidemiology, Biostatistics and Prevention Institute University of Zurich Zurich Switzerland; 2 Geriatric Education and Research Institute Singapore Singapore; 3 Division of Medical Information Sciences University Hospitals of Geneva Geneva Switzerland

**Keywords:** patient-generated health data, personal health information, consumer health information, primary prevention, health promotion, telemedicine, mobile health, medical informatics, eHealth

## Abstract

**Background:**

Digital innovations continue to shape health and health care. As technology socially integrates into daily living, the lives of health care consumers are transformed into a key source of health information, commonly referred to as patient-generated health data (PGHD). With chronic disease prevalence signaling the need for a refocus on primary prevention, electronic PGHD might be essential in strengthening proactive and person-centered health care.

**Objective:**

This study aimed to review and synthesize the existing literature on the utilization and implications of electronic PGHD for primary disease prevention and health promotion purposes.

**Methods:**

Guided by a well-accepted methodological framework for scoping studies, we screened MEDLINE, CINAHL, PsycINFO, Scopus, Web of Science, EMBASE, and IEEE Digital Library. We hand-searched 5 electronic journals and 4 gray literature sources, additionally conducted Web searches, reviewed relevant Web pages, manually screened reference lists, and consulted authors. Screening was based on predefined eligibility criteria. Data extraction and synthesis were guided by an adapted PGHD-flow framework. Beyond initial quantitative synthesis, we reported narratively, following an iterative thematic approach. Raw data were coded, thematically clustered, and mapped, allowing for the identification of patterns.

**Results:**

Of 183 eligible studies, targeting knowledge and self-awareness, behavior change, healthy environments, and remote monitoring, most literature (125/183, 68.3%) addressed weight reduction, either through physical activity or nutrition, applying a range of electronic tools from socially integrated to full medical devices. Participants generated their data actively (100/183, 54.6%), in combination with passive sensor-based trackers (63/183, 34.4%) or entirely passively (20/183, 10.9%). The proportions of active and passive data generation varied strongly across prevention areas. Most studies (172/183, 93.9%) combined electronic PGHD with reflective, process guiding, motivational and educational elements, highlighting the role of PGHD in multicomponent digital prevention approaches. Most of these interventions (110/183, 60.1%) were fully automatized, underlining broader trends toward low-resource and efficiency-driven care. Only a fraction (47/183, 25.6%) of studies provided indications on the impact of PGHD on prevention-relevant outcomes, suggesting overall positive trends, especially on vitals (eg, blood pressure) and body composition measures (eg, body mass index). In contrast, the impact of PGHD on health equity remained largely unexplored. Finally, our analysis identified a list of barriers and facilitators clustered around data collection and use, technical and design considerations, ethics, user characteristics, and intervention context and content, aiming to guide future PGHD research.

**Conclusions:**

The large, heterogeneous volume of the PGHD literature underlines the topic’s emerging nature. Utilizing electronic PGHD to prevent diseases and promote health is a complex matter owing to mostly being integrated within automatized and multicomponent interventions. This underlines trends toward stronger digitalization and weaker provider involvement. A PGHD use that is sensitive to identified barriers, facilitators, consumer roles, and equity considerations is needed to ensure effectiveness.

## Introduction

### Background

The emergence of digital health innovations is expected to continue shaping the organization and delivery of health services [1,2]. As technology integrates into multiple domains of daily living, its potential for disrupting health systems and societal impact rapidly expands [1,3]. On an individual level, the uptake of smart and wearable technology pushes the boundaries of self-quantification and generates novel opportunities to monitor and promote health [2,4]. These developments gradually transform the lives of health care consumers into key health information sources. The output is commonly referred to as patient-generated health data (PGHD) [5].

### Electronic and Patient-Generated Health Data

A landmark whitepaper by the US Office of the National Coordinator for Health Information Technology defines PGHD as health-related information created by patients or their designees outside traditional health care contexts [6]. The current emergence of PGHD can be partially attributed to 2 dominant digitalization trends: the societal integration of mobile phones and the growing health-related use of Web-based media [7-9]. Preinstalled mobile phone applications and integrated sensors enable the continuous measurement of physical, mental, social, and environmental health parameters, whereas online platforms and social media increasingly become a place for health communication and depositories of large data volumes [7,8,10]. These trends gradually transform consumers from passive recipients to active agents of their health [1]. Acknowledging the wide social integration of mobile devices, the generation of one’s own health information might be a potential way to engage medically underserved populations and close long-lasting inequity gaps [11].

### Digital and Proactive Prevention

As the prevalence of chronic diseases continues to rise, many health care systems face unprecedented challenges that deem it necessary to refocus on prevention [12]. Without disregarding the challenges of electronic PGHD, generating one’s own health information might provide an incentive for behavioral change, facilitating health literacy and knowledge exchange [13,14]. Data sharing can in turn trigger personalized feedback, customized health plans, and tailored persuasive health promotion techniques [14,15]. In other words, PGHD can contribute to proactive, informed, and prevention-focused health systems, as well as personalized and collaborative care [16-18]. Despite those benefits, systematically and comprehensively synthesized knowledge on the use of such data for primary disease prevention and health promotion purposes seems to be lacking.

### Objectives

Our overarching objective targets the synthesis of the literature on the overall utilization of electronic PGHD for primary disease prevention and health promotion purposes. Specific objectives include (1) providing an overview of applied PGHD types and tools, as well as their aims, purposes, and contexts, (2) exploring health care consumer, provider, and technology responsibilities, as well as potential interactions among them, and (3) synthesizing broader implications of electronic PGHD on health outcomes and equity.

## Methods

### Methodological Framework

#### Overview

Our methodology was guided by Arksey and O’Malley’s framework for scoping studies and Levac, Colquhoun, and O’Brien’s conceptual extensions [19,20]. We divided our approach accordingly into 6 steps, described separately in the following sections. Study quality and evidence strength assessment fall beyond the aims of a scoping review and were not performed [19]. A detailed description of our methodological and conceptual background has been published elsewhere [21]. Protocol deviations and their justifications are provided in Multimedia Appendix 1.

#### Step 1: Identifying the Research Question

Our research question was formed by an iterative process by getting acquainted with the literature, identifying existing evidence gaps, as well as by regular exchange and expert consultation. Our question consisted of the 3 previously mentioned study objectives across the underlying dimensions of (1) data generation and collection, (2) sharing or communication, (3) interpretation, and (4) utilization. We narrowed the definition of electronic PGHD to data generated by consumer-facing means, excluding information that was collected through standardized, provider-driven methods, such as predefined questionnaires [22], which is justified on the very nature of primary disease prevention and health promotion that ideally requires an active patient. Similarly, we used the term health care consumer, instead of patient, as our target population consists of individuals free of any International Classification of Disease–coded conditions. Nonetheless, we kept the *patient* in PGHD, as that is an already coined term. The term provider is conceptualized as any professional who is responsible for offering health and well-being–related services.

#### Step 2: Identifying Relevant Studies

With the support of a specialized librarian and preliminary literature review, we developed an extensive and purposively sensitive search strategy, applied to 7 electronic databases that included MEDLINE, Cumulative Index to Nursing and Allied Health Literature, PsycInfo, Scopus, Web of Science, EMBASE, and Institute of Electrical and Electronics Engineers Digital Library. The searches were conducted on February 1, 2018. We additionally hand-searched 5 key electronic journals and 4 gray literature sources, complemented by Web searches, using the first 10 page results of 3 engines and thorough screenings of 6 relevant Web pages. Our last research steps consisted of (1) the manual reference list screening of all eligible studies and (2) author consultations, requesting input on potentially missed or unpublished work. A more detailed description of our study identification strategy is provided in previously published protocol [21].

The full search strategy and search terms are provided in Multimedia Appendix 2.

#### Step 3: Study Selection

A total of 2 members of the research team (VN and PL) independently conducted a screening of the titles and abstracts, as well as full text screening against a set of predefined eligibility criteria (Textbox 1). Not fulfilling all conditions below led to exclusion.

**Study eligibility criteria.**

- Addresses electronic patient-generated health data (PGHD), as defined by this review, and additionally does the following:
  - Includes at least one sentence on the electronic PGHD tool or type.
  - Includes at least one sentence on how these are used or created.
  - Addresses PGHD that are available in a digital format at the point of utilization for intended health-related purposes, irrespective of the generation process.
- Has a main focus on primary prevention and health promotion and falls within one the following domains:
  - Preventing initial occurrence of disease in healthy or high-risk individuals.
  - Mitigating risk in healthy or high-risk individuals.
  - Promoting existing health.
- Describes, explores, and analyzes some form of health care consumer and provider involvement, where *provider* can be a technology or a human provider.
- Addresses an adult population.
- Is a primary study published in English or German between January 1, 2003, and January 31, 2018.

#### Step 4: Charting the Data

Data extraction was conducted by 2 reviewers (VN and PL), guided by a predefined, flexible data extraction form, to capture the review’s objectives and corresponding research questions. The final form was refined and validated through consultation and expert feedback. Impact data were broadly extracted in terms of significance and direction. Equity data were extracted according to Cochrane Equity Group recommendations [23]. The 2 reviewers (VN and PL) initially tested the form on a random sample of 5 studies, following immediate comparisons and adjustments [20,24]. Owing to the large volume of the included literature, extractions were divided among the 2 reviewers. To minimize subjective bias, 27.3% (50/183) of all completed data extraction forms were randomly selected for validation by a third reviewer who added comments and amendments. Recommended changes were discussed and integrated with consensus.

#### Step 5: Collating, Summarizing, and Reporting the Results

The whole process, including data charting (Step 4), was guided by an adapted PGHD flow framework, provided in Multimedia Appendix 3 [6,21]. Our adapted version visualizes the flow of PGHD from the consumer, passing through intermediaries (technology or health care provider) and back to the consumer in the form of prevention and health promotion impact. Initial synthesis was quantitative, aiming to provide a descriptive summary of study and participant characteristics, as well as the extent, scope, and nature of the existing literature. Further synthesis was qualitative and followed an iterative thematic approach [20]. Raw data were coded, thematically clustered, and transformed into conceptual maps that facilitated the identification of patterns. The entire process, including screening (Step 3) and data extraction (Step 4), was conducted in Covidence (Cochrane), a Web-based systematic reviewing tool, and DistillerSR (Evidence Partners), a multi-functional software for all types of literature reviews. Reporting is based on the Preferred Reporting Items for Systematic Reviews and Meta-Analyses (PRISMA) statement [25].

#### Step 6: Consultation

An external PGHD expert was consulted twice during the conceptualization stage who provided content-related feedback. A total of 3 stakeholders, a provider-partner (physician) and 2 consumer-partners, were consulted during the final manuscript preparation stages to ensure that our interpretations were relevant and understandable.

## Results

### Summary

**Figure 1:**
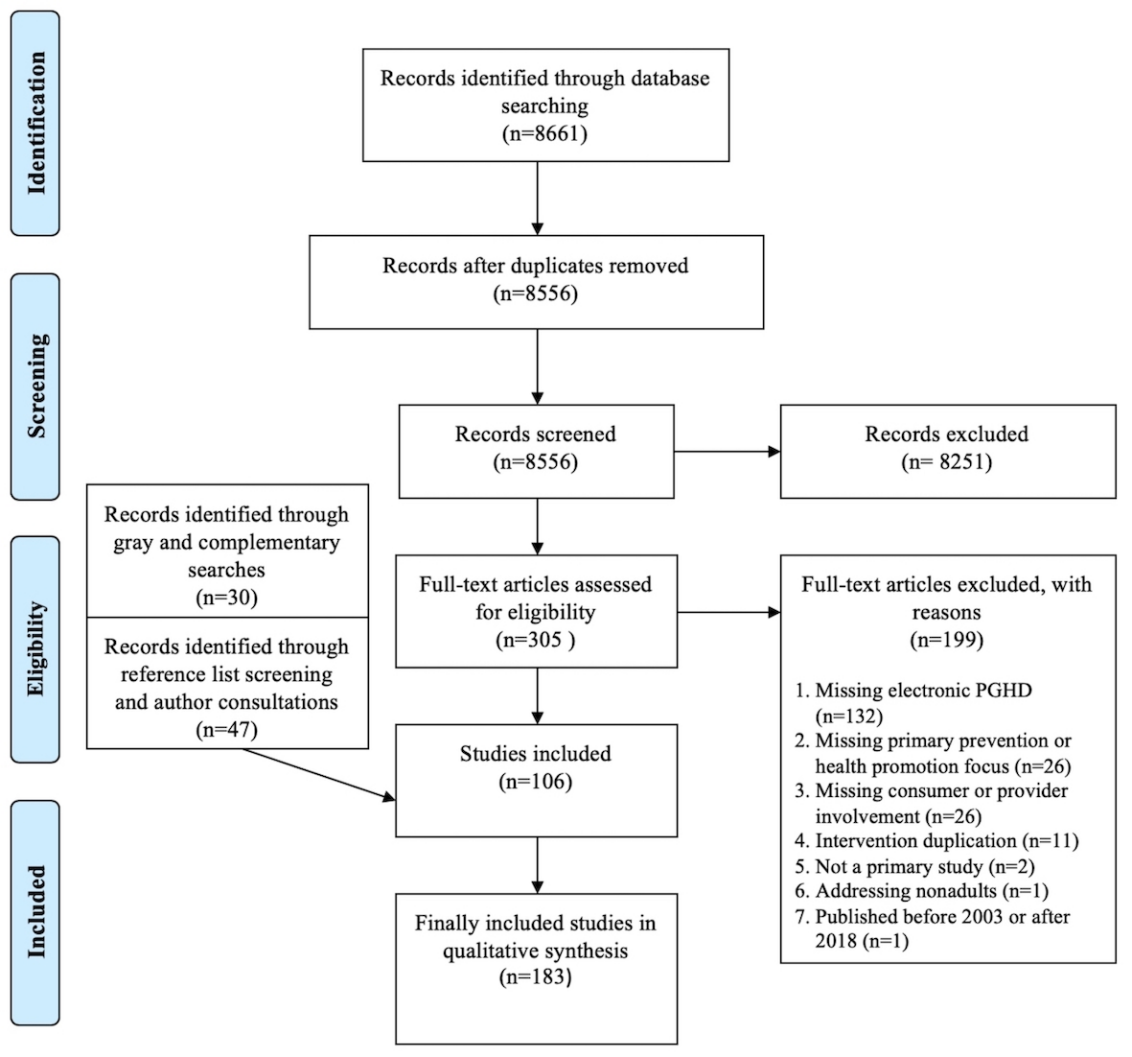
Preferred Reporting Items for Systematic Reviews and Meta-Analyses flow chart. PGHD: patient-generated health data.

The deduplicated database search resulted in 8556 citations, which were screened by titles and abstracts. Full-text appraisal was deemed eligible for 305 studies, of which 199 did not fulfill our inclusion criteria. In total, the electronic database searches yielded 106 included studies. Interrater agreement reached 84% (42/50) (k=0.411) for a sample of 50 studies during title and abstract screening and 93% (14/15) (k=0.636) for a sample of 15 studies during full-text review. Complementary searches, including hand searching and searching gray literature sources, led to the inclusion of 30 studies, whereas reference list screenings and author consultations led to 47 additional studies, resulting in a total of 183 inclusions. A list of all excluded references at full-text screening, including justifications, is provided in Multimedia Appendix 4. A list of included studies and their extracted study characteristics are provided in Multimedia Appendices 5 and 6, respectively. The PRISMA flow chart [25] in Figure 1 summarizes the whole study process.

### Study Characteristics

Most eligible studies were published as scientific journal articles (n=162), followed by sections of conference proceeding collections (n=13) and published theses (n=8). With an average of 22 studies per year, most were published from 2011 onward, whereas the number of publications averaged to around 6 studies a year between 2003 and 2010. More than half of the studies were conducted in North America (n=107), with 105 from the United States and 2 from Canada. European research followed with 38 studies, most of which were conducted in the United Kingdom. The remaining were conducted in Australia and New Zealand (n=18), Asia (n=13), and the Middle East (n=1). A total of 6 studies had an international scope. Randomized controlled trials constituted most of the applied methodologies (n=93), followed by quantitative nonrandomized approaches (n=47), mixed-method designs (n=30), and purely qualitative methodologies (n=13). The majority aimed to demonstrate effectiveness and efficacy (n=99), followed by mixed and purely exploratory aims (n=52), whereas less than a quarter explored feasibility and acceptability of interventions (n=32). The duration of identified studies ranged from a single examination to up to 2 years. More detailed information on study characteristics, including percentages, is provided in Multimedia Appendix 5. Participant characteristics are summarized in Multimedia Appendix 7.

### Electronic Patient-Generated Health Data—Enabled Prevention Areas and Aims

The most commonly addressed prevention area was weight management, primarily in the form of physical activity and nutrition, which consists of 68.3% (125/183) of the identified literature. This is followed by 12.0% (22/183) of studies with a broader focus on health and well-being. These studies did not exclusively focus on one prevention area and included combinations of chronic and infectious disease, as well as mental health. About 7.7% (14/183) of the literature addressed cardiometabolic health, whereas 7.1% (13/183) focused on substance use prevention, targeting tobacco and alcohol. Healthy aging, such as prevention of falls, cognitive decline, and bone health, was the subject of 6 studies (6/183, 3.3%), followed by 2 studies on breastfeeding (2/183, 1.1%) and 1 study on skin cancer prevention (1/183, 0.5%). Multimedia Appendix 8 provides a list of included studies grouped by prevention areas.

We continued our analysis by synthesizing information on the aims of generating and sharing PGHD for primary disease prevention and health promotion purposes. We identified that enabling health consumers to generate their own health information aims at (1) promoting healthy behavior (142/183, 77.5%), (2) increasing health knowledge and self-awareness (120/183, 65.5%), (3) enabling healthy environments (60/183, 32.7%), and (4) enhancing remote monitoring (20/183, 10.9%). Most studies (134/183, 73.2%) targeted 2 or more of those aims. A similar pattern was observed within prevention areas, with health behavior change and knowledge gain being the most commonly addressed aims. This was not the case for substance use prevention, where enabling healthy environments outweighed knowledge gain. Not all studies adhered strictly to those aims, with 21.3% (39/183) deviating from purely preventive purposes and additionally using PGHD as outcome measures, for example, to quantify the effects of interventions and for secondary analyses. Table 1 describes the 4 identified aims and provides examples from the literature.

**Table 1:** Description and examples of patient-generated health data aims.

Aim	Description	Example from the literature
Increase health knowledge and self-awareness	Increase in knowledge and cognition about one’s health, well-being, and behavior, with no particular focus on how to translate this knowledge into action and concrete behavior	Participants record dietary intake and receive weekly feedback with summaries on their fruit, vegetable, and junk food intake [26]
Promote healthy behavior	Help translate one’s knowledge into action, behavior change, and skill development, targeting health improvement and maintenance	Participants record dietary intake and receive nutritional feedback and additional individual dietary targets, recipes, and a meal plan for achieving those [27]
Enable healthy environments	Enable environments and contexts that facilitate health and well-being	Participants record physical activity in a digital partnership with family members or friends, creating an environment of healthy social pressure and support [28]
Enhance remote monitoring	Enable the remote monitoring of individual health and well-being parameters, by health care and wellness providers	Participants record blood pressure, blood glucose, weight, and body fat at home and sent data electronically to medical professional who monitors and provides personalized physical activity plans [29]

### The Role of Consumers

Successful prevention undoubtedly requires a clear definition of health care consumer responsibilities. Our analysis identified 3 broad consumer roles. The first consisted of passive PGHD generation (20/183, 10.9%), in which consumers used sensor-based devices to automatically collect and transmit information. Such an approach was predominantly applied in physical activity, weight loss, and overall health and well-being, capturing data that did not require manual entries, such as step counts, heart rate, and sleep quality. The more common second and third roles consisted of fully (100/183, 54.6%) or partially (63/183, 34.4%) active consumers, requiring occasional to regular actions. Active consumer involvement is key for capturing data that are not easily captured automatically, such as consumed meals and the quantity of smoked cigarettes. The term *partially active* describes all approaches that involve both active and passive data generation. That includes anything that is not exclusively sensor-based, nor exclusively dependent on manual entries. Partially active data generation was highly prevalent in prevention areas that often require the simultaneous collection of multiple heterogenous measures (eg, steps, food intake, blood pressure and blood glucose), which was the case in weight loss, physical activity, nutrition, and cardiometabolic health. Table 2 summarizes consumer roles and provides illustrative literature examples. Table 3 provides the spread of consumer roles across identified prevention areas.

**Table 2:** Patient-generated health data–related consumer roles and examples.

Consumer roles		Examples from the literature
**Data generation roles**		
	Fully active data generation	Take picture of meal and optionally add descriptions, visit website to add further contextual information [30]
	Partially active data generation	Manually record stress levels and automatically capture data by wearing heart monitor [31]
	Passive data generation	Carry mobile phone an physical activity monitor that generates PGHD^a^ automatically [32]
**Data sharing roles**		
	Low-intensity data sharing	PGHD automatically stored on mobile phone based database and automatically transmitted in an encrypted manner [33]
	High-intensity data sharing	Share data manually from monitors to website (directly or via a docking station) [34-35]

^a^PGHD: patient-generated health data.

Data generation is often followed by data sharing to third parties or across devices and storage locations. Information on data sharing was provided by about 73.2% (134/183) of the literature. We defined high-intensity sharing as any transmission of PGHD that requires concrete consumer action. High-intensity sharing was applied in 91 studies (91/183, 49.7%). Half of those (39/91, 43%) indicated more demanding actions requiring the active transfer of PGHD to external devices (eg, external computer) or storage locations (eg, server and website). In contrast, low-intensity sharing describes the automatic transmission of PGHD, which was applied in 43 (43/183, 23.5%) studies. We did not identify any difference of distribution between higher or lower sharing intensity across most prevention areas, except for cardiometabolic health and weight loss (Table 3). In cardiometabolic health, most studies described high-intensity data sharing. This can be attributed to the frequent use of less connected devices, such as blood pressure monitors and glucometers. An opposite trend was observed in weight loss interventions that tended to adopt low-intensity sharing, which could be attributable to the sophistication and good interoperability of fitness trackers. Among the studies (n=163/183) with fully or partially active consumers, 137 provided clear information on the frequency of PGHD sharing. Out of those, 110 (110/137, 80.3%) described daily and 27 (27/137, 19.7%) less than daily sharing frequency.

**Figure 2:**
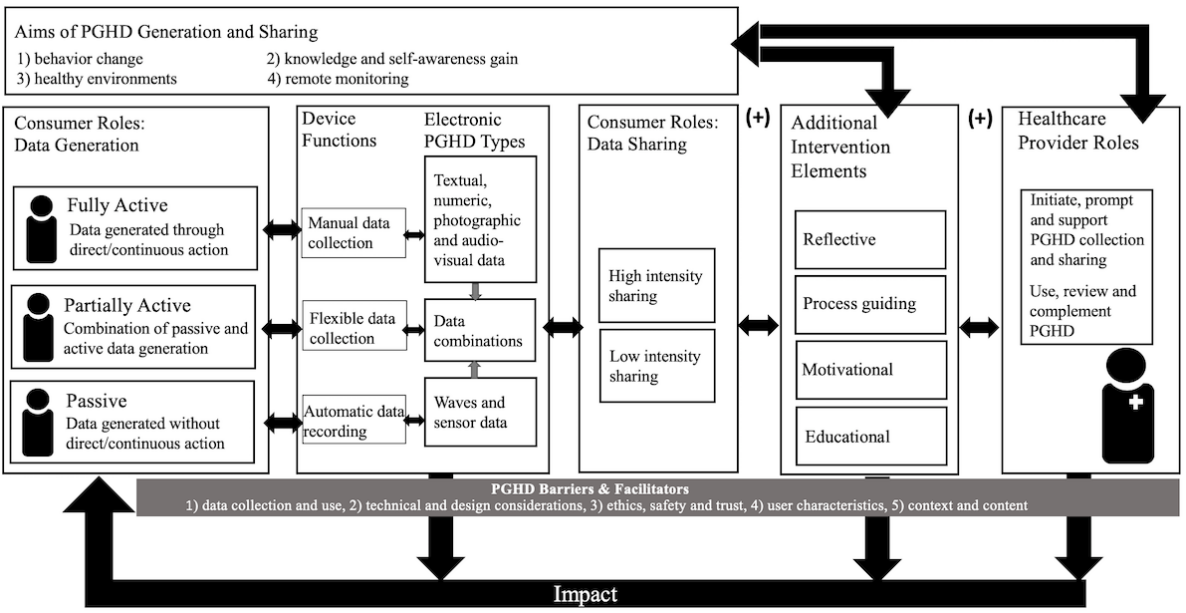
Enriched conceptual framework of electronic patient-generated health data (PGHD) flow and use for primary disease prevention and health promotion.

On the basis of the initial framework by Shapiro et al, we developed an extended and more comprehensive conceptual framework [6,21]. With consumer roles as a starting point, our framework visualizes the generation and flow of electronic PGHD and is adapted to the context of primary disease prevention and health promotion. The framework, illustrated in Figure 2, organizes the study’s key results and visualizes identified patterns.

Our enriched framework shows that the 3 identified consumer roles are linked to different PGHD tools, ultimately creating different clusters of data. Although we identified interventions stopping at that level (single-component), the majority entailed additional intervention components (multicomponent), with and without human provider involvement. Thus, prevention and health promotion impact can be achieved at 3 levels, as the lower arrows indicate. Across the different elements, from the consumer to the provider, 5 common areas of barriers and facilitators can inhibit or promote the effective use of electronic PGHD. The framework additionally visualizes the link between identified PGHD aims and additional intervention components, as well as the involvement of health care providers. This framework fulfills the function of providing a simplified process overview, ultimately fostering a better understanding of PGHD utilization across prevention areas. All framework components are detailed throughout the results section.

**Table 3:** Distribution overview of key themes across prevention areas.

Prevention areas		Weight control, physical activity, nutrition (n=125), n (%)	Overall health and well-being (n=21), n (%)	Cardio- metabolic health (n=14), n (%)	Substance use (smoking and alcohol; n=14), n (%)	Healthy aging (n=6), n (%)	Breastfeeding (n=2), n (%)	Skin cancer (n=1), n (%)
**Consumer roles (data generation)^a^**								
	Active data generation	65 (52.0)	11 (52)	4 (29)	14 (100)	3 (50)	2 (100)	1 (100)
	Partially active data generation	46 (36.8)	7 (33)	9 (64)	0 (0)	1 (17)	0 (0)	0 (0)
	Passive data generation	14 (11.2)	3 (15)	1 (7)	0 (0)	2 (33)	0 (0)	0 (0)
**Consumer roles (data sharing)^a^**								
	High-intensity data sharing	43 (34.4)	9 (43)	8 (58)	4 (29)	1 (17)	0 (0)	1 (100)
	Low-intensity data sharing	51 (40.8)	8 (38)	3 (21)	4 (29)	2 (33)	0 (0)	0 (0)
	Unclear or not described	31 (24.8)	4 (19)	3 (21)	6 (42)	3 (50)	2 (100)	0 (0)
**Health care provider roles**								
	Support and motivate PGHD^b^	2 (1.6)	3 (14)	0 (0)	0 (0)	0 (0)	0 (0)	0 (0)
	Review and analyze PGHD	23 (18.4)	1 (5)	5 (36)	3 (21)	1 (17)	1 (50)	0 (0)
	Support and Motivate PGHD and review and analyze PGHD combined	12 (9.6)	0 (0)	4 (29)	1 (7)	0 (0)	0 (0)	0 (0)
	Non-PGHD-related involvement	14 (11.2)	2 (10)	1 (7)	0 (0)	0 (0)	0 (0)	0 (0)
	No involvement at all	74 (59.2)	15 (71)	4 (28)	10 (72)	5 (83)	1 (50)	1 (100)
**Utilized hardware^c^**								
	Nonhealth-related (eg, phone)	104 (83.2)	18 (86)	12 (86)	14 (100)	5 (83)	2 (100)	1 (100)
	Health-related (eg, pedometer)	65 (52.0)	12 (87)	10 (71)	0 (0)	2 (33)	0 (0)	0 (0)
	Medical (eg, glucometer)	2 (1.6)	3 (14)	5 (36)	5 (36)	0 (0)	0 (0)	0 (0)
**Additional intervention components^a,c^**								
	Reflective	113 (90.4)	16 (76)	13 (93)	11 (79)	5 (83)	2 (100)	0 (0)
	Process guiding	99 (79.2)	9 (43)	13 (93)	12 (86)	5 (83)	1 (50)	1 (100)
	Motivational	88 (70.4)	8 (38)	7 (50)	13 (93)	4 (67)	0 (0)	1 (100)
	Educational	84 (67.2)	6 (29)	10 (71)	10 (71)	2 (33)	2 (100)	0 (0)

^a^Consumer roles are described in patient-generated health data–related consumer roles and examples table. Additional intervention components are defined in detail in descriptions of intervention components.

^b^PGHD: patient-generated health data.

^c^Studies were assigned to multiple hardware and additional intervention component categories, for which rows do not add up to 100%.

### Electronic Patient-Generated Health Data Tools, Their Functions, and Data Types

Prevention-targeted PGHD were generated through 3 broad types of hardware, often used in combination. The first included nonhealth-related products that are mostly well integrated into daily living (157/183, 85.8%), such as computers and mobile phones. The second entailed health-related devices that are less societally integrated (90/183, 49.2%), such as pedometers and heart rate monitors. The third included more specialized medical devices (15/183, 8.2%), such as glucometers and blood pressure monitors. Beyond manual and automatic PGHD collection, their most common functions included the provision of additional intervention components, such as goal setting and reminders, analysis and visualization of data, provision of feedback, sharing and storage of PGHD, and communication and interaction with third parties. Another key function was the provision of cues and visualizations, such as using color schemes, pictures, avatars, and other virtual elements to support the interpretation of PGHD. Table 3 provides the distribution of utilized hardware across prevention areas. Multimedia Appendix 9 offers more detailed information on identified tools and their functions. Most studies (175/183, 95.6%) have additionally described the use of software, such as apps, mobile- and Web-based programs, Web-based platforms and websites, social media and forums, device-installed software, and email and text messaging. The remaining 4.4% (8/183) did not explicitly mention any utilized software.

The identified electronic PGHD were categorized in 4 broad types. Most studies (78/183, 42.6%) addressed textual or numerical data, requiring manual entry and an active consumer. This was followed by waves or signals (22/183, 12.0%) that did not require manual collection and audiovisual (video) (4/183, 2.2%) as well as photographic (2/183, 1.1%) PGHD, which again required an active user. Textual or numerical data were mostly used in weight control, substance use prevention, and healthy aging. Photographic data were applied in healthy eating, whereas audiovisual PGHD were commonly utilized in smoking and alcohol prevention. Waves and signals were primarily applied in weight control, well-being, cardiometabolic health, and healthy aging. Finally, almost half of the studies used 2 or more forms of digital PGHD (77/183, 42.1%), with the most common combinations being that of textual or numerical with waves or signals (58/77, 75%) and textual or numerical with photographic data (7/77, 9%). Textual or numerical with waves or signals was used across the spectrum of health domains from weight control to diabetes prevention and usually included initial sensor data that were then manually recorded by users. Textual or numerical with photographic data was applied in dietary interventions, where users took pictures of their meals and added descriptions.

### Electronic Patient-Generated Health Data Use: Additional Intervention Components

In 172 (172/183, 93.9%) of the identified studies, PGHD were embedded in larger multicomponent preventive interventions. Our analysis identified 4 overarching components to which PGHD were combined with, categorized as (1) reflective, (2) process guiding, (3) motivational, and (4) educational. Their descriptions and examples are provided in Textbox 2. Most interventions entailed at least one (162/183, 88.5%) purely reflective component, whereas 77.6% (142/183) included at least one process guiding component. Motivational components were included in 67.2% (123/183) of all interventions, with social support (eg, online peer interaction) constituting 33.3% (61/183) of those. Finally, educational components were identified in 63.4% (116/183) of all studies. A distinct element of educational components was the provision of support for making sense of one’s health data, through *a-priori* training or instructions, as well as targeted immediate or retrospective feedback. Table 3 provides the distribution of additional intervention components across prevention areas. The boundaries between those 4 components were not fixed, and each study was often assigned to more than 1 category. Most of these components (147/172, 85.5%) were entirely or partially tailored to individual participants, whereas a relatively smaller proportion (25/172, 14.5%) were predominantly nontailored or unclear. The overlap between identified aims for collecting PGHD and additional interventions components (eg, educational components and the aim of knowledge enhancement, motivational components, and aim of behavior change) suggested that underlying reasons for generating and using PGHD for preventive purposes might influence and be influenced by the availability of these additional intervention components. Additional intervention components are defined in detail in Textbox 2.

**Descriptions of intervention components.**

1. Reflective: All intervention components that are based on simple feedback of generated patient-generated health data (PGHD), with no additional educational information on how these are to be interpreted and applied. Examples include PGHD reports and summaries, as well as access to unstructured data.
2. Process guiding: All intervention components that aim to provide general support on the generation of PGHD, the use of technology, the compliance to intervention guidelines, and the response to problems arising from these processes. They include technical advice, instructions on when and how to collect and share, and problem-solving advice. Guidance in understanding and applying PGHD falls out this category’s scope (see point 4. Educational).
3. Motivational: All intervention components that are based on techniques that target the motivation of users to collect PGHD and apply those for healthy behavior changes. They include the provision of rewards and incentives, persuasion techniques, goal setting, reminders, motivational feedback, social support, as well as entertainment elements, such as gamification.
4. Educational: All intervention components that go beyond the simple feedback of generated PGHD (reflective), being attached to additional information that targets knowledge and skill enhancement, as well as knowledge testing. In contrast with process guiding, this category focuses on understanding and applying PGHD. They include the provision of newsletters, in-person counseling, remote coaching, educational podcasts, quizzes, and knowledge tests. Technical guidance on the generation and share of PGHD falls out of this category’s scope (see point 2. Process guiding).

The integration and utilization of electronic PGHD varied across additional intervention components. In combination with reflective components, PGHD were mostly used for self-referencing, such as the visualization of progress over time, enabling users to track individual health goals. In the context of motivational components, PGHD were repeatedly utilized to enable social comparison, such as contrasting data to normative or peer-generated values, often generating certain social pressure for healthier lifestyles. When integrated with educational components, PGHD were used to inform and provide individualized recommendations and counseling, aligned to the progress and capabilities of individual participants or participant subgroups. Finally, in combination with process guiding components, PGHD were key to identifying individual challenges, allowing for tailored and problem-focused support, while ensuring that adherence to intervention guidelines (eg, dietary or exercise plans) was monitored.

### The Role of Health Care Providers

Less than half of the literature described the role of health care providers in the implementation (73/183, 39.9%) of interventions and only 30.6% (56/183) involved providers that had clearly designated PGHD-related responsibilities. The remaining proportion of the literature (110/183, 60.1%) addressed predominantly automatic programs. The proportion of studies without provider involvement was larger across all prevention areas, except for those on cardiometabolic health and breastfeeding promotion. Health care providers included an array of professionals, including physicians, nurses, dieticians, psychologists, health consultants, fitness experts, and trainers. Our thematic analysis identified 2 main clusters of PGHD-related provider roles. The first role (5/55, 9%) is that of a supporter, including the prompting, overseeing, and motivating of PGHD use, which was primarily found in weight control, nutrition and well-being interventions. The second role (34/55, 62%) is that of a reviewer, consisting of analyzing PGHD to inform counseling, personalize advise, conduct remote monitoring, and complement medical data, which was common in weight control, nutrition, cardiometabolic health, and substance use prevention. In 31% (17/55) of the studies, mainly in weight control, cardiometabolic health, and substance use prevention, providers held both roles simultaneously. In addition, we identified that provider-consumer interactions predominantly occurred remotely (36/73, 49%), either via the PGHD tool itself (eg, data collection website) or through other supporting channels (eg, email), both in a synchronous (eg, telephone) or asynchronous fashion (eg, forums). In-person interactions were less common (14/73, 19%) and more often combined with remote elements (17/73, 23%). One study (1/73, 1%) involved no direct interaction with consumers, whereas 5 (5/73, 7%) lacked clear interaction descriptions. Our findings additionally suggested that the involvement of health care providers was linked to the previously described PGHD aims, as one of those, namely the aim of enhanced remote monitoring, inevitably relies on data review by a provider. Table 3 provides the distribution of provider roles across prevention areas.

### Patient-Generated Health Data Implications: Health Impact and Equity

Assessed prevention-relevant outcomes were broadly categorized into: (1) vitals and body composition measures (eg, body mass index, blood pressure, blood glucose, and heart rate), (2) behavioral change (eg, physical activity, eating habits, and lifestyle factors), and (3) knowledge change (eg, health literacy and awareness). About a quarter of the identified literature (47/183, 25.7%) provided indications on the potential impact of PGHD on preventive outcomes. These studies either had PGHD as a distinct or single component in one of their intervention arms (13/47, 28%), or as a part of multicomponent interventions (34/47, 72%), with sections that explored the associations between PGHD (eg, adherence to data collection) and outcomes. The majority explored implications on vitals and body composition-related outcomes (37/47, 79%). Most of those studies reported statistically significant beneficial trends (n=27), followed by nonsignificant effects (n=8) and mixed results (n=4). Outcomes in health behavior were less commonly addressed (15/47, 32%) and provided no clear tendencies, with an equal number of studies providing statistically significant beneficial (n=4) and nonsignificant (n=4) trends, as well as a relatively large proportion of unclear or mixed results (n=3). Health knowledge outcomes were the least commonly (2/47, 4%) addressed, with one study reporting nonsignificant associations between PGHD and health knowledge and one reporting mixed results. Most of these studies included active (27/47, 57%) and partially active consumers (8/47, 17%), whereas only one study entailed passive consumers (1/47, 2%). For the studies with active and partially active user engagement, a proportionally equal number of them reported statistically significant, mixed, and nonstatistically significant results. One study that included passive consumers did not provide enough information to be meaningfully compared.

A larger proportion of the literature (98/183, 53.6%) addressed interventions with multiple components and did not entail analyses on the relationship between PGHD components and prevention outcomes. Although their results could not be directly linked to PGHD, the overall picture suggested beneficial trends, with 23% (22/98) providing almost entirely positive results. Mixed results were indicated by 69% (68/98) of studies, almost all of which included at least one significantly positive outcome. A smaller proportion of interventions (8/96, 8%) did not identify beneficial effects at all. The remaining part of the literature (38/183, 20.8%) focused on feasibility and usability results instead, which is not reported in further detail here.

Considering equity as an important outcome for all health interventions, we extracted information linked to implications for subgroups that are commonly divided by health inequalities, as defined by the Cochrane Equity Group [23]. About 46.5% of studies (85/183) addressed equity by referring to the digital divide and literacy inequalities, by addressing the limitations of homogeneous study samples that primarily consisted of advantaged subgroups (eg, white, highly educated), by focusing on underserved populations, and by exploring patterns across sociodemographic variables. Approximately 7.7% of the literature (14/183) provided detailed analyses across subgroups divided by sex (12/14), age (6/14), race (4/14), education (4/14), income (2/14), and place of residence (1/14). Most of those indicated either no or unclear differential effects, whereas 2 indicated better intervention effects for younger participants, one for white non-Hispanic individuals and one for higher educated participants.

### Barriers and Facilitators of Electronic Patient-Generated Health Data Use

About 89.6% (164/183) of studies provided information on potential barriers and facilitators of electronic PGHD. Both barriers and facilitators were clustered around 5 recurring themes: (1) data collection and use, (2) technical and design considerations, (3) ethics, safety, and trust, (4) user characteristics, and (5) context and content. Data collection and use (127/164, 77.4%) addressed the levels of ease, difficulty, and burden of electronic PGHD generation, the adaptability of data collection to user needs, and associated resource demands (eg, time, costs). Technicalities and design (84/164, 51.2%) covered the functional maturity of PGHD technology, the facilitating role of mobile and interoperable devices, as well as the importance of dynamic, user-appealing, and simple designs. Ethics, safety, and trust (55/164, 33.5%) entailed barriers and facilitators around privacy, trustworthiness, credibility, and reliability. The category of user characteristics (72/164, 43.9%) highlighted consumer-related elements, such as digital literacy, knowledge, sociodemographic determinants, and overall attitudes toward PGHD technologies. Finally, the last category of content and context (148/164, 90.2%) included elements around contextual resources, such as PGHD support and interaction with providers. It additionally addressed the role of technology and intervention content, such as the combination of PGHD with other behavior change communication techniques. Textboxes 3 and 4 provide a list of identified barriers and facilitators across those 5 themes.

**Barriers of electronic patient-generated health data and the number of studies reporting barriers in each domain.**

Data collection and use (n=49):

- Burdensome data collection
- Inflexible data entry
- Retrospective data entry: incentive to manipulate data
- Unstructured data: information overload
- Automatized recording: feeling of no control over data
- Costly

Technicalities and design (n=39):

- Immature or nonfunctional
- Unappealing design
- Nonuser-friendly functions

Ethics, safety, and trust (n=32):

- Privacy and security concerns
- Nontrustworthy patient-generated health data (PGHD) tools and data
- Sociocultural resistances
- Low-quality and unreliable PGHD

User characteristics (n=38):

- Low digital literacy and no previous experience
- Negative attitudes toward PGHD
- Mismatch with daily life routines
- Nonperceived usefulness
- Sociodemographics (eg, young age, low education)

Content and context (n=44):

- Missing data interpretation and general support
- Missing in-person contact
- Missing or too frequent reminders
- Missing provider resources to evaluate PGHD
- Missing (financial) incentives or rewards
- Missing or insensitive feedback
- Unrealistic goals
- Nonengaging environment (eg, no social support)

**Facilitators of electronic patient-generated health data and the number of studies reporting facilitators in each domain.**

Data collection and use (n=78):

- Simple and low-effort data collection
- Highly flexible data entry
- Retrospective data entry: incentive to correct data
- Time-efficient and intuitive data output
- Automatized recording: noninterference with daily life
- Free or low cost

Technicalities and design (n=45):

- Technically mature and interoperable
- User-engaging and appealing design
- Dynamic design: interactive and modifiable
- Mobile

Ethics, safety, and trust (n=23):

- Credible patient-generated health data (PGHD) tools
- Trustworthy, reliable, and complete PGHD
- Processes that do not invade privacy

User characteristics (n=34):

- Digital literacy and previous experience
- Preexisting motivation and readiness to use PGHD
- Self-efficacy
- Perceived PGHD usefulness and relevance

Content and context (n=104):

- Available guidance and support
- Available human interaction
- Sensitive reminders that do not disturb
- Data visualizations and summaries
- Motivating rewards and (financial) incentives
- Immediate, sensitive, and motivating feedback
- Realistic goal setting
- Social support (eg, peer interactions)
- Content and context personalization
- Enabled data access, ownership, and control
- Fun elements (eg, gamification)
- Novel elements (eg, geofence triggered support)

## Discussion

### Overview

Our review described a large and dynamically emerging volume of the literature on the use of electronic PGHD for primary disease prevention and health promotion purposes. Beyond quantity, the literature manifested large methodological and thematic heterogeneity, adding to the topic’s conceptual complexity. Our results enabled the development of an enriched conceptual framework (Figure 2) and thus a better understanding of the process between generating PGHD and finally utilizing them for preventive and health promoting action.

### Principal Findings and Comparison With Previous Work

The identified literature predominantly focused on weight control, through physical activity and nutrition, which was consistent with previous reviews that addressed digital health interventions across prevention areas [36-38]. In line with the variety of existing digital approaches to primary disease prevention [39-40], our results indicated that electronic PGHD target multiple dimensions, including: (1) health knowledge and self-awareness, (2) behavior change, (3) healthy environments, and (4) remote monitoring. Overall, we identified 4 types of prevention-targeted interventions entailing electronic PGHD: (1) automatic, single component, (2) automatic multicomponent interventions, (3) single component, and (4) multicomponent interventions that are not fully automatic, including health care provider involvement (Figure 2). A single component denotes that PGHD is the main and only prevention element, whereas a multicomponent describes interventions with at least 1 additional non-PGHD element.

Our thematic analysis identified certain recurring patterns of PGHD generation. We broadly classified consumer roles as passive, partially active, and fully active and identified that the proportions of these vary across prevention areas. Acknowledging that consumer roles are closely linked to PGHD types, we found that certain prevention areas are being dominated by 1 or 2 types of PGHD. On one hand, weight control, alcohol and smoking prevention, and overall health and well-being seemed to be mostly addressed by technologies that require the manual collection of textual or numerical data, while on the other hand, cardiometabolic disease prevention was primarily addressed by a combination of PGHD types that require a mix of active and passive data generation. In contrast, entirely passive data generation was only identified for weight control, overall health and well-being, cardiometabolic health, and healthy aging. Although not focused on prevention, a review by Vagesna et al [41] identified similar patterns, where weight was monitored by computerized systems (manual data entry) and metabolic conditions by a combination of multiple technologies.

Considering that the sophistication and reliability of PGHD technology varies across prevention areas, these patterns are expected. To be adequately targeted and well-informed, prevention often requires very specific consumer action and PGHD input. On one hand, for certain areas, such as addiction prevention or dietary intake, this input is entirely behavioral and not easily captured automatically. This includes the exact number of smoked cigarettes and consumed alcohol drinks, the type of consumed drinks, the percentage of alcohol content in each drink or the portions of consumed meals, all of which currently cannot be reliably or cost effectively collected by sensor-based devices, while on the other hand, sophisticated and highly commercialized fitness trackers are increasingly being improved to reliably capture certain activities and bodily functions, such as physical exercise and heart rate. Exercise-based weight loss, well-being promotion (eg, sleep quality), and healthy aging (eg, fall prevention) are prevention areas in which such devices can be applied to, which explains the prevalence of passive PGHD generation. In between the two extremes, there are prevention approaches that inherently require combinations of measures, such as in diabetes prevention (eg, dietary intake and physical activity), which in turn allow for a partially active and partially passive generation of PGHD.

Linking consumer roles to identified barriers and facilitators suggests some conflicting dynamics. Passive PGHD generation might be less burdensome, but may also lead to lower consumer engagement. Conversely, active generation involves more effort but may simultaneously trigger higher user motivation. In their review on wearable monitoring technology, Baig et al [42] described part of these challenges, such as that passive application of technology might counteract user engagement and acceptance. Another review linked the intensity of consumer responsibilities to intervention effectiveness, reporting that programs with active consumers (eg, manual data input) were more successful [43]. To fully understand the most effective use of electronic PGHD for prevention, these patterns suggest that is important to further explore the interactions between PGHD types and their demands on consumers.

The relatively large proportion of studies that described automatic prevention systems, constituting 60.1% (110/183) of the identified literature, underlines a broader trend toward low-recourse and efficiency-driven care [44]. As expected, this was not the case for areas that traditionally rely on close patient-provider relationships, such as cardiometabolic health and breastfeeding. The remaining 39.9% (73/183) of the literature indicated 2 main health care provider clusters: (1) supporting PGHD collection and subsequently, (2) reviewing or using data for preventive practice. If fully automatized, those tasks were transferred to consumers, or the technology itself. A scoping review on the preventive use of smart devices by Petit and Cambon [45] described consumer responsibilities as a key literature aspect, in which patients gained expert roles and became equal agents of their own health care. Interalia, this has potential implications on data interpretation that we have identified as a recurring concept across studies. Previous research demonstrated the importance of PGHD interpretation and its wider implications [46], with missing sense-making skills and the fear of self-interpretation mentioned as key challenges to personal health information use. Our findings reflected that importance, as a major proportion of the literature directly or indirectly addressed interpretability by describing various approaches to fostering correct data understanding.

Most identified studies integrated electronic PGHD within multicomponent interventions, either complementing or facilitating other intervention components (eg, enabling self-reflection, facilitating social comparison, informing counseling, and directing guidance). A systematic review on the use of technology for weight reduction identified a similar trend, with 19 out of 27 studies combining PGHD with counseling feedback [47]. These trends highlight the value of PGHD for complex digital prevention, especially when not entailing health care providers. In addition, a previous scoping review by Lentferink et al identified that using PGHD to personalize intervention components (eg, goal setting) seemed to be a distinct element of highly effective studies [43].

Summarizing the results of single-component interventions, exploratory analyses (eg, associations between PGHD and health outcomes), and overall effects of multicomponent interventions, the overall directions suggest a predominantly positive PGHD impact on prevention. These trends are expected, considering the existing evidence on the association between monitoring one’s own health and preventive outcomes [48-50]. On the contrary, trends on the impact of PGHD on health inequity gaps were not as clear, as only 7.7% (14/183) of the literature reported on differential effects across subgroups. The limited information and methodological ambiguity around subgroup analyses [51] did not allow for confident equity statements. Equity reviews from other fields, such as population and primary-care-based physical activity interventions reported on similar challenges, with only 19% (17/87) and 14.0% (24/171) of primary studies providing information on differential distribution of effects [52,53]. This is problematic, considering the potential implications of the digital divide, which is transforming from a divide on technology access to a divide on digital literacy and skills [54]. If we fail to adequately address equity, we risk limiting the preventive benefits of technology to those few who have the resources and skills to use them appropriately. Nonetheless, it is encouraging that almost half of the literature directly or indirectly mentioned equity, which indicates that many researchers are aware of the importance of ethics in digital health research.

Finally, the existence of conflicting barriers and facilitators highlighted the currently emerging nature and potential knowledge gaps of the topic. When do reminders become disturbances in one’s daily life and when are they the key to ensuring prevention adherence? Are automatic and simple data collection methods preferred by consumers because of being less burdensome, or do they counteract user engagement and motivation? Do financial rewards act purely as incentives to collect data and adhere to preventive guidelines, or could they become incentives for data falsification? Are PGHD tools that allow for retrospective data entries beneficial because of added flexibility, or do they add to the risk of data manipulation? Although these uncertainties may be indicators of an emerging topic that requires more research, they might also be the result of electronic health and prevention complexity. Neither digitalization nor prevention are static or fixed phenomena [55]. They ultimately depend on interactions and contexts, for which single answers may be difficult to find. Nonetheless, the richness and range of identified barriers and facilitators indicate that the preventive use of electronic PGHD is sensitive to many factors, be it the way data are collected, the context in which they are collected, the personal characteristics of users, as well as ethical and technological considerations.

### Limitations

Despite our rigorous methodological approach, our study is subjected to some limitations. First, although PGHD may be key throughout the continuum of care, our review’s scope is restricted to primary disease prevention and health promotion. As such, our overall findings might not be applicable to the domains of treatment, disease management, and rehabilitation. This scope is narrower than defined in our protocol and has been chosen for practical and conceptual reasons. Retaining a broader scope would have led to an unmanageable volume of the literature and challenges in meaningfully synthesizing the results. Second, the chosen definition of electronic PGHD, which emphasized the aspect of patient’s control, led to the exclusion of standardized and more provider-driven approaches. Broadening the definition might provide a better understanding of PGHD-based prevention within health care contexts and the interaction of such data streams with health care provider infrastructures. Third, the variety and evolvement of definitions and terms to describe PGHD, as well as prevention, might have led to missing out a few terms and the associated literature. To compensate for that, we conducted thorough hand searches, reference list screenings, and author consultations. Finally, our scoping methodology and heterogeneous output did not allow for robust synthesis and comparison of effects.

### Implications and Future Research

#### Overall Implications

The patterns we identified may support users, patients, and providers in understanding the complexity of utilizing electronic PGHD for prevention purposes. Beyond technical maturity, providers need to consider the wider implications that data collection might have on patients and consumers, such as its interference with daily living, personal beliefs, and digital literacy. Users and providers need to be sensitive to ethical and trust concerns, while ensuring that the PGHD environments are motivating and supportive enough to facilitate adherence and successful prevention.

#### Future Research

Scoping reviews are often conducted to assess the feasibility of conducting a full systematic review [19]. On the basis of the large and very heterogeneous literature volume, we believe that conducting a full systematic review, while retaining a similarly broad scope, will be conceptually difficult. We, therefore, suggest (1) a narrower scope, (2) a careful *a priori* consideration of a PGHD definition, as that differed across the literature and may impact the review’s results, (3) a careful selection of search terms, and (4) a preparatory literature scan to identify all terms appropriate to the chosen scope, as the terminology is vast and evolving. Beyond systematic reviews, future research should target evidence on best possible combinations of electronic PGHD with other behavioral change techniques (eg, feedback, goal setting, and peer interaction). Finally, future research should aim to capture how barriers and facilitators vary across contexts, while addressing the wider implications of PGHD-based prevention on the functioning of health systems and health equity.

### Conclusions

Our review provides a comprehensive picture of the literature on electronic PGHD use for primary disease prevention and health promotion purposes, enabling a broader identification of processes and patterns. The high heterogeneity in the scope and content of identified studies underlines the topic’s emerging nature. This is reflected by the variety of identified PGHD-generating technologies, resulting in diverse data types and different consumer responsibilities. Utilizing electronic PGHD to prevent disease and promote health is a complex matter. In the literature, this complexity arises from electronic PGHD being mostly integrated into multicomponent and automatized interventions, limiting our ability to assess their individual preventive impact, and underlining an overall trend toward larger consumer responsibility. The broad set of identified barriers and facilitators, some being conflicting, highlights the need for a holistic understanding of such enabling factors, as well as for a stronger focus on ethical challenges, which is currently lacking.

## Appendix

Multimedia Appendix 1 Protocol deviations and justifications.

Multimedia Appendix 2 Search strategy and utilized keywords.

Multimedia Appendix 3 Adapted conceptual framework of electronic patient-generated health data flow.

Multimedia Appendix 4 List of studies excluded at full-text appraisal, with reasons.

Multimedia Appendix 5 List and characteristics of included studies.

Multimedia Appendix 6 Extracted study characteristics.

Multimedia Appendix 7 Participant characteristics.

Multimedia Appendix 8 List of included studies, grouped by prevention area.

Multimedia Appendix 9 Patient-generated health data tools and their functionalities.

## Abbreviations

PGHD: patient-generated health data
PRISMA: Preferred Reporting Items for Systematic Reviews and Meta-Analyses
