# Supplementary material for: Electronic Patient-Generated Health Data to Facilitate Disease Prevention and Health Promotion: Scoping Review
Source: J Med Internet Res. 2019 Oct 14;21(10):e13320. doi: 10.2196/13320 (PMC6914107; doi:10.2196/13320)
Supplement: Multimedia Appendix 1 [file jmir_v21i10e13320_app1.pdf]

Multimedia Appendix: Protocol deviations and justifications

Deviation 1: Focus on primary prevention instead of primary and secondary prevention

The reason for focusing our scope to primary prevention is based on practical and conceptual arguments. Firstly, preliminary searches, retaining a broader scope and including secondary and tertiary prevention yielded a vast amount of literature, deeming data synthesis impractical and unfeasible. Secondly, the dynamics driving different prevention levels, as commonly classified (primary, secondary, tertiary prevention) differ enough to pose conceptual challenges of synthesis within a single review, for which focusing on one prevention level was deemed more appropriate.

Deviation 2: Non-duplicate data extraction

The volume of included literature was larger than expected, for which a fully duplicate extraction posed time and human power requirements that went beyond available resources. Nonetheless, we aimed to mitigate any potential resulting bias by having a third reviewer validating about 50% of all data extractions.
