# Supplementary material for: Electronic Patient-Generated Health Data to Facilitate Disease Prevention and Health Promotion: Scoping Review
Source: J Med Internet Res. 2019 Oct 14;21(10):e13320. doi: 10.2196/13320 (PMC6914107; doi:10.2196/13320)
Supplement: Multimedia Appendix 2 [file jmir_v21i10e13320_app2.pdf]

Multimedia Appendix: Search strategy and utilized keywords

#1	((patient NEXT/1 (reported OR shared) NEAR/3 (data OR information)):ti,ab) OR (((consumer OR people OR user OR person*) NEXT/1 reported NEAR/6 (health OR medical OR clinical) NEXT/1 (information OR data)):ti,ab) OR ((connected NEXT/1 (health OR medicine)):ti,ab)
#2	((patient NEXT/3 portal):ti,ab) OR (((electronic OR digital OR online OR web* OR internet) NEXT/3 'health diary'):ti,ab)
#3	'electronic patient record'/exp OR 'electronic medical record'/exp OR 'electronic health record'/de OR 'telemedicine'/exp OR (((personal OR user OR consumer OR electronic OR online OR digital OR web OR internet OR computer) NEAR/1 (medical OR health OR clinical) NEXT/1 record):ti,ab) OR ((patient* NEXT/3 record):ti,ab
#4	((patient OR consumer OR people OR user OR person* OR self*) NEXT/1 (generated OR reported OR shared)):ti,ab
#5	self:ti,ab OR oneself:ti,ab OR himself:ti,ab OR herself:ti,ab OR personal*:ti OR connected:ti,ab OR ((personal* NEXT/3 (health* OR medicine* OR care OR manag* OR monitor*)):ti,ab)
#6	#4 OR #5
#7	#3 AND #6
#8	#1 OR #2 OR #7
#9	promot*:ti OR prevent*:ti OR improve*:ti OR (((health OR patient) NEAR/3 (educat* OR communicat* OR advocacy OR literacy OR behaviour OR behavior OR status)):ti) OR (((disease OR health OR personalized) NEXT/3 manag*):ti) OR ((self NEXT/1 (manag* OR monitor*)):ti)
#10	#8 AND #9
#11	'health promotion'/exp OR 'health literacy'/exp OR 'health education'/exp OR 'disease management'/exp OR 'health behavior'/exp OR 'health status'/exp OR ((health NEAR/1 (promot* OR prevent* OR educat* OR communicat* OR advocacy OR literacy OR behaviour OR status)):ti,ab) OR ((disease NEAR/1 manag*):ti,ab) OR (((disease OR medicine) NEAR/3 prevent*):ti,ab) OR ((self NEXT/1 (manag* OR monitor*)):ab)
#12	'devices'/exp OR 'internet'/exp OR 'information processing'/exp OR (((electronic* OR mobile OR smart) NEXT/3 (tool* OR watch* OR device* OR gadget* OR bracelet* OR pager* OR monitor*)):ti,ab) OR (((mobile OR cell OR smart) NEXT/3 phone):ti,ab) OR tablet*:ti,ab OR iphone*:ti,ab OR ipad*:ti,ab OR smartphone*:ti,ab OR wearable*:ti,ab OR app:ti,ab OR apps:ti,ab OR application*:ti,ab OR ((technol* NEAR/3 (consumer OR patient OR user)):ti,ab)
#13	innovat*:ti,ab
#14	#12 OR #13
#15	#8 AND #11 AND #14
#16	#8 AND #11 AND #12
#17	#10 OR #15
#18	((patient NEXT/1 generated NEAR/3 (data OR information)):ti,ab) OR (((consumer OR people OR user OR person*) NEXT/1 generated NEAR/6 (health

	OR medical OR clinical) NEXT/1 (information OR data)):ti,ab) OR ((connected NEXT/1 (health OR medicine)):ti,ab)
#19	(connected NEXT/1 (health* OR medicine OR treat* OR monitor* OR care*)):ti,ab
#20	#17 OR #18 OR #19
#21	'electronic patient record'/exp OR 'electronic medical record'/exp OR 'electronic health record'/de OR 'telehealth'/exp OR 'medical informatics'/exp OR (((personal OR user OR consumer OR electronic OR online OR digital OR web OR internet OR computer) NEAR/1 (medical OR health OR clinical) NEXT/1 record):ti,ab) OR ((patient* NEXT/3 record):ti,ab) OR (((electronic OR digital OR mobile OR tele) NEXT/1 (health OR care OR monitoring)):ti,ab) OR 'e health':ti,ab OR 'm health':ti,ab OR 'e care':ti,ab OR 'm care':ti,ab OR 'e monitoring':ti,ab OR 'm monitoring':ti,ab OR 'internet of things':ti,ab OR telemedicine:ti,ab OR ((health NEXT/1 (it OR 'information technology')):ti,ab**)**
#22	#6 AND #21
#23	#1 OR #2 OR #22
#24	#9 AND #23
#25	#11 AND #14 AND #23
#26	#18 OR #19 OR #24 OR #25
	Date: 12.12.2017<br>Filters: Adult, Human, English & German, 2003 – to present

Keywords used for more general searches, applied to: IEEE Digital Library, hand-searches, grey literature sources, web engines and webpages.

1. Self-monitoring AND health
2. Self-tracking AND health
3. Self-monitoring AND health promotion
4. Self-tracking AND health promotion
5. Self-monitoring AND prevention
6. Self-tracking AND prevention
7. Self-monitoring AND wellness
8. Self-tracking AND wellness
9. Patient-generated health
10. Patient generated data
11. Patient generated information
