# Supplementary figures and images for: Electronic Patient-Generated Health Data to Facilitate Disease Prevention and Health Promotion: Scoping Review
Source: J Med Internet Res. 2019 Oct 14;21(10):e13320. doi: 10.2196/13320 (PMC6914107; doi:10.2196/13320)

Multimedia Appendix: Adapted conceptual framework of electronic PGHD flow [6, 21]

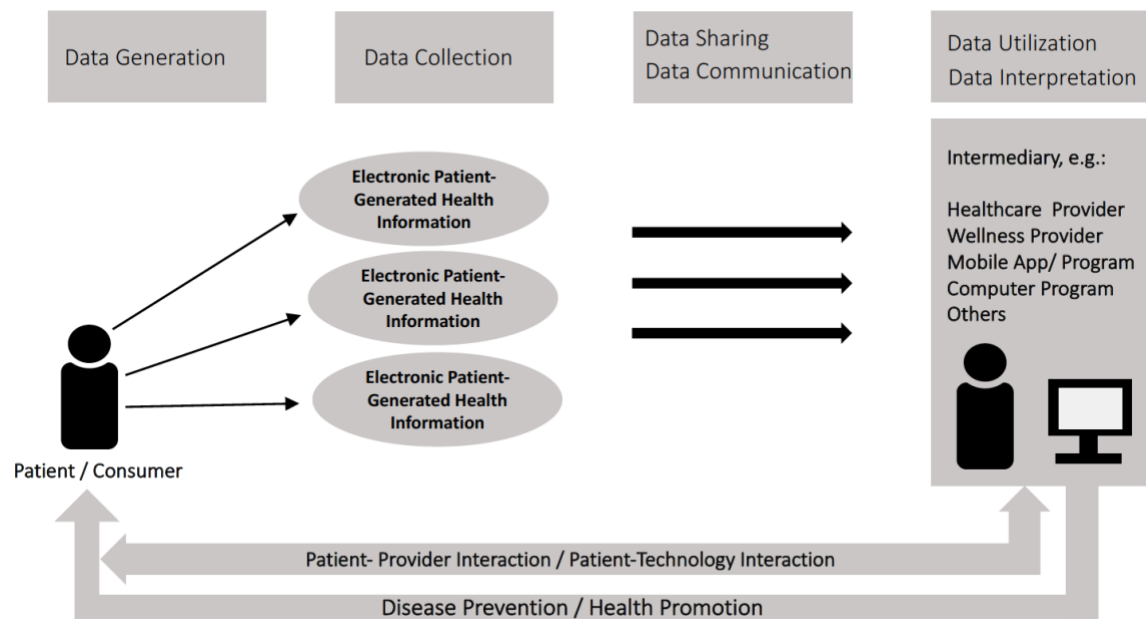

Supplement: Multimedia Appendix 3 [file jmir_v21i10e13320_app3.pdf]
