# Supplementary material for: Electronic Patient-Generated Health Data to Facilitate Disease Prevention and Health Promotion: Scoping Review
Source: J Med Internet Res. 2019 Oct 14;21(10):e13320. doi: 10.2196/13320 (PMC6914107; doi:10.2196/13320)
Supplement: Multimedia Appendix 4 [file jmir_v21i10e13320_app4.pdf]

Multimedia Appendix: List of studies excluded at full-text appraisal, with reasons

Exclusion Code	Reason
1	Does not address electronic PGHD in accordance with study's definition and in sufficient in detail, as outline by study's eligibility criteria
2	Does not address primary prevention and health promotion in accordance with the study's defined scope
3	Does not describe patient involvement/tasks or provider responsibilities/ or technology functions
4	Does not address an adult population
5	Is not written in English or German
6	Is not a primary study
7	Is an intervention duplication
8	Is not published between 01.01.2003 and 31.01.2018

List of Excluded Studies with Exclusion Codes

Hwang, K. O., Ning, J., Trickey, A. W., & Sciamanna, C. N. (2013). Website usage and weight loss in a free commercial online weight loss program: Retrospective cohort study. *Journal of Medical Internet Research*, *15*(1), 1–10. Exclusion Reason: 3

Choe, E. K., Lee, B., Kay, M., Pratt, W., & Kientz, J. A. (2015). SleepTight : Low-burden , Self-monitoring Technology for Capturing and Reflecting on Sleep Behaviors. In *UBICOMP*. Osaka. Exclusion Reason: 7

Banos, O., Villalonga, C., Garcia, R., Saez, A., Damas, M., Holgado-Terriza, J. A., … Rojas, I. (2015). Design, implementation and validation of a novel open framework for agile development of mobile health applications. *Biomedical Engineering Online*, *14 Suppl 2*, S6–S6. Exclusion Reason: 3

Merom, D., Rissel, C., Phongsavan, P., Smith, B. J., Kemenade, C. Van, Brown, W. J., & Bauman, A. E. (2007). Promoting Walking with Pedometers in the Community. *American Journal of Pre*, *32*(4), 290–297. Exclusion Reason: 1

Partridge, S. R., McGeechan, K., Bauman, A., Phongsavan, P., & Allman-Farinelli, M. (2017). Improved confidence in performing nutrition and physical activity behaviours mediates behavioural change in young adults: Mediation results of a randomised controlled mHealth intervention. *Appetite*, *108*, 425–433. Exclusion Reason: 1

Livingstone, K. M., Celis-Morales, C., Navas-Carretero, S., San-Cristoba, R., MacReady, A. L., Fallaize, R., … Mathers, J. C. (2016). Effect of an Internet-based, personalized nutrition randomized trial on dietary changes associated with the Mediterranean diet: The Food4Me Study. *American Journal of Clinical Nutrition*, *104*(2 PG-288-297), 288–297. Exclusion Reason: 1

Krist, A. H., Peele, E., Woolf, S. H., Rothemich, S. F., Loomis, J. F., Longo, D. R., & Kuzel, A. J. (2011). Designing a patient-centered personal health record to promote preventive care. *BMC Medical Informatics And Decision Making*, *11*, 73. Exclusion Reason: 1

Espinoza, J., Chen, A., Orozco, J., Deavenport-Saman, A., & Yin, L. (2017). Effect of personal activity trackers on weight loss in families enrolled in a comprehensive behavioral family-lifestyle intervention program in the federally qualified health center setting: A randomized controlled trial. *Contemporary Clinical Trials Communications*, *7*(June), 86–94. Exclusion Reason: 6

Stuckey, M., Fulkerson, R., Read, E., Russell-Minda, E., Munoz, C., Kleinstiver, P., & Petrella, R. (2011). Remote monitoring technologies for the prevention of metabolic syndrome: the Diabetes and Technology for Increased Activity (DaTA) study. *Journal of Diabetes Science and Technology*, *5*(4), 936–944. Exclusion Reason: 7

Sheehan, B., James Lucero, R., & James, R. (2015). Initial Usability and Feasibility Evaluation of a Personal Health Record-Based Self-Management System for Older Adults Recommended Citation) "Initial Usability and Feasibility Evaluation of a Personal Health Record-Based Self-Management System for Ol. *Geriatric Nursing Commons*, *3*(2), 5–7. Exclusion Reason: 1, 3

Mummah, S. A., Mathur, M., King, A. C., Gardner, C. D., & Sutton, S. (2016). Mobile Technology for Vegetable Consumption: A Randomized Controlled Pilot Study in Overweight Adults. *JMIR MHealth and UHealth*, *4*(2), e51. Exclusion Reason: 7

Cohen, D. J., Keller, S. R., Hayes, G. R., Dorr, D. A., Ash, J. S., & Sittig, D. F. (2016). Integrating Patient-Generated Health Data Into Clinical Care Settings or Clinical Decision-Making: Lessons Learned From Project HealthDesign. *JMIR Human Factors*, *3*(2), e26. Exclusion Reason: 2

Homma, S., Imamura, H., Nakamura, T., Fujimura, K., Ito, Y., Maeda, Y., & Kaneko, I. (2016). A comparative study on the effectiveness of one-way printed communication versus videophone interactive interviews on health promotion. *Journal Of Telemedicine And Telecare*, *22*(1 PG-56-63), 56–63. Exclusion Reason: 2

Turner-McGrievy, G. M., Beets, M. W., Moore, J. B., Kaczynski, A. T., Barr-Anderson, D. J., & Tate, D. F. (2013). Comparison of traditional versus mobile app self-monitoring of physical activity and dietary intake among overweight adults participating in an mHealth weight loss program. *Journal Of The American Medical Informatics Association: JAMIA*, *20*(3), 513–518. Exclusion Reason: 1

McTigue, K. M., Conroy, M. B., Hess, R., Bryce, C. L., Fioeillo, A. B., Fischer, G. S., … Simkin-Silverman, L. R. (2009). Using the Internet to Translate an Evidence-Based Lifestyle Intervention into Practice. *Telemedicine and E-Health*, *15*(9), 851–858. Exclusion Reason: 2

Sharit, J., Idrees, T., Andrade, A. D., Anam, R., Karanam, C., Valencia, W., … Ruiz, J. G. (2018). Use of an online personal health record's Track Health function to promote positive

lifestyle behaviors in Veterans with prediabetes. *Journal of Health Psychology*, *23*(5), 681–690. Exclusion Reason: 2

Sidman, C. L., Corbin, C. B., & Masurier, G. Le. (2004). Promoting physical activity among sedentary women using pedometers. *Research Quarterly for Exercise and Sport*, *75*(2), 122–129. Exclusion Reason: 1

Lee, W., Chae, Y. M., Kim, S., Ho, S. H., & Choi, I. (2010). Evaluation of a mobile phone-based diet game for weight control. *Journal of Telemedicine and Telecare*, *16*(5), 270–275. Exclusion Reason: 2

Carter, M. C., Burley, V. J., & Cade, J. E. (2017). Weight Loss Associated With Different Patterns of Self-Monitoring Using the Mobile Phone App My Meal Mate. *JMIR MHealth and UHealth*, *5*(2), e8. Exclusion Reason: 7

Vanwormer, J. J., Martinez, A. M., Benson, G. A., Crain, A. L., Martinson, B. C., Cosentino, D. L., … Vanwormer, J. J. (2009). Telephone Counseling and Home Telemonitoring : The Weigh By Day Trial. *American Journal Of Health Behavior*, *33*(4), 445–454. Exclusion Reason: 2

Ozok, A. A., Wu, H., Garrido, M., Pronovost, P. J., & Gurses, A. P. (2014). Usability and perceived usefulness of personal health records for preventive health care: A case study focusing on patients' and primary care providers' perspectives. *Applied Ergonomics*, *45*(3 PG-613-628), 613–628. Exclusion Reason: 1

Kerns, J. W., Krist, A. H., Longo, D. R., Kuzel, A. J., & Woolf, S. H. (2013). How patients want to engage with their personal health record: A qualitative study. *BMJ Open*, *3*(7). Exclusion Reason: 3

Herring, S. J., Cruice, J. F., Bennett, G. G., Rose, M. Z., Davey, A., & Foster, G. D. (2016). Preventing excessive gestational weight gain among African American women: A randomized clinical trial. *Obesity (Silver Spring, Md.)*, *24*(1 PG-30-36), 30–36. Exclusion Reason: 1

Rickard, N., Arjmand, H.-A., Bakker, D., & Seabrook, E. (2016). Development of a Mobile Phone App to Support Self-Monitoring of Emotional Well-Being: A Mental Health Digital Innovation. *JMIR Mental Health*, *3*(4), e49. Exclusion Reason: 1

Turner-McGrievy, G., & Tate, D. (2011). Tweets, Apps, and Pods: Results of the 6-month Mobile Pounds Off Digitally (Mobile POD) randomized weight-loss intervention among adults. *Journal of Medical Internet Research*, *13*(4), 1–14. Exclusion Reason: 1

Burke, L. E., Conruy, M. B., Sereika, S. M., Elci, O. U., Styn, M. A., Acharya, S. D., … Glanz, K. (2011). The Effect of Electronic Self-Monitoring on Weight Loss and Dietary Intake: A Randomized Behavioral Weight Loss Trial. *Obesity*, *19*(2). Exclusion Reason: 7

Ma, J., Yank, V., Xiao, L., Lavori, P. W., Wilson, S. R., Rosas, L. G., & Stafford, R. S. (2013). Translating the diabetes prevention program lifestyle intervention for weight loss into

primary care: A randomized trial. *JAMA Internal Medicine*, *173*(2), 113–121. Exclusion Reason: 2

Rasche, P., Mertens, A., Bröhl, C., Theis, S., Seinsch, T., Wille, M., … Knobe, M. (2017). The "Aachen fall prevention App" - a Smartphone application app for the self-assessment of elderly patients at risk for ground level falls. *Patient Safety In Surgery*, *11*, 14. Exclusion Reason: 1

Conroy, M. B., Bryce, C. L., McTigue, K. M., Tudorascu, D., Gibbs, B. B., Comer, D., … Fischer, G. S. (2017). Promoting weight maintenance with electronic health record tools in a primary care setting: Baseline results from the MAINTAIN-pc trial. *Contemporary Clinical Trials*, *54*, 60–67. Exclusion Reason: 3

Robroek, S. J. W., Brouwer, W., Lindeboom, D., Oenema, A., & Burdorf, A. (2010). Demographic, behavioral, and psychosocial correlates of using the website component of a worksite physical activity and healthy nutrition promotion program: a longitudinal study. *Journal of Medical Internet Research*, *12*(3), 1–16. Exclusion Reason: 7

Williams, R. L., Wood, L. G., Collins, C. E., & Callister, R. (2014). Effectiveness of weight loss interventions - is there a difference between men and women: A systematic review. *Obesity Reviews*, *16*(2), 171–186. Exclusion Reason: 6

Lau, A. Y. S., Proudfoot, J., Andrews, A., Liaw, S. T., Crimmins, J., Arguel, A., & Coiera, E. (2013). Which bundles of features in a web-based personally controlled health management system are associated with consumer help-seeking behaviors for physical and emotional well-being? *Journal of Medical Internet Research*, *15*(5), 1–19. Exclusion Reason: 1

Mattila, E., Korhonen, I., Salminen, J. H., Ahtinen, A., Koskinen, E., Särelä, A., … Lappalainen, R. (2010). Empowering citizens for well-being and chronic disease management with wellness diary. *IEEE Transactions On Information Technology In Biomedicine: A Publication Of The IEEE Engineering In Medicine And Biology Society*, *14*(2 PG-456-463), 456–463. Exclusion Reason: 3

Chung, L. M. Y., Law, Q. P. S., Fong, S. S. M., & Chung, J. W. Y. (2014). Electronic dietary recording system improves nutrition knowledge, eating attitudes and habitual physical activity: A randomised controlled trial. *Eating Behaviors*, *15*(3), 410–413. Exclusion Reason: 7

Naveh-Deutsch, N., Ish-Shalom, S., Rozen, G. S., & Bitterman, N. (2007). Interactive computer nutrition system for elderly. *Gerontechnology*, *6*(4), 236–240. Exclusion Reason: 1

Magoc, D., Tomaka, J., & Bridges-arzaga, A. (2011). Using the Web to Increase Physical Activity in College Students. *American Journal Of Health Behavior*, *35*(2), 142–154. Exclusion Reason: 1

Krist, A. H., Woolf, S. H., Bello, G. A., Sabo, R. T., Longo, D. R., Kashiri, P., … Cohn, J. (2014). Engaging Primary Care Patients to Use a Patient- Centered Personal Health Record. *Annals of Famil*, *12*(5). **Exclusion Reason: 1**

Collins, C. E., Morgan, P. J., Jones, P., Fletcher, K., Martin, J., Aguiar, E. J., … Callister, R. (2010). Evaluation of a commercial web-based weight loss and weight loss maintenance program in overweight and obese adults: a randomized controlled trial. *BMC Public Health*, *10*(1), 669. **Exclusion Reason: 3**

Krist, A. H., Aycock, R. A., Etz, R. S., Devoe, J. E., Sabo, R. T., Williams, R., … Woolf, S. H. (2014). MyPreventiveCare: implementation and dissemination of an interactive preventive health record in three practice-based research networks serving disadvantaged patients--a randomized cluster trial. *Implementation Science: IS*, *9*, 181. **Exclusion Reason: 3**

Morgan, P. J., Collins, C. E., Plotnikoff, R. C., Cook, A. T., Berthon, B., Mitchell, S., & Callister, R. (2011). Efficacy of a workplace-based weight loss program for overweight male shift workers: The Workplace POWER (Preventing Obesity Without Eating like a Rabbit) randomized controlled trial. *Preventive Medicine*, *52*(5 PG-317-325), 317–325. **Exclusion Reason: 7**

Lau, A. Y. Y. S., Dunn, A. G., Mortimer, N., Gallagher, A., Proudfoot, J., Andrews, A., … Coiera, E. (2013). Social and self-reflective use of a Web-based personally controlled health management system. *Journal Of Medical Internet Research*, *15*(9), e211–e211. **Exclusion Reason: 1**

Allman-Farinelli, M., Partridge, S. R., McGeechan, K., Balestracci, K., Hebden, L., Wong, A., … Bauman, A. (2016). A Mobile Health Lifestyle Program for Prevention of Weight Gain in Young Adults (TXT2BFiT): Nine-Month Outcomes of a Randomized Controlled Trial. *JMIR Mhealth And Uhealth*, *4*(2), e78–e78. **Exclusion Reason: 1**

Dorough, A. E., Winett, R. A., Anderson, E. S., Davy, B. M., Martin, E. C., & Hedrick, V. (2014). DASH to wellness: emphasizing self-regulation through e-health in adults with prehypertension. *Health Psychology: Official Journal Of The Division Of Health Psychology, American Psychological Association*, *33*(3), 249–254. **Exclusion Reason: 2**

Ma, J., Xiao, L., & Blonstein, A. C. (2013). Measurement of self-monitoring web technology acceptance and use in an e-health weight-loss trial. *Telemedicine Journal And E-Health: The Official Journal Of The American Telemedicine Association*, *19*(10), 739–745. **Exclusion Reason: 2**

Staffileno, B. A., Tangney, C. C., Fogg, L., & Darmoc, R. (2015). Making Behavior Change Interventions Available to Young African American Women: Development and Feasibility of an eHealth Lifestyle Program. *The Journal Of Cardiovascular Nursing*, *30*(6 PG-497-505), 497–505. **Exclusion Reason: 2**

Kim, J. (2014). Analysis of health consumers' behavior using self-tracker for activity, sleep, and diet. *Telemedicine Journal And E-Health: The Official Journal Of The American Telemedicine Association*, *20*(6), 552–558. **Exclusion Reason: 1, 2**

Turner-McGrievy, G. M., & Tate, D. F. (2014). Are we sure that Mobile Health is really mobile? An examination of mobile device use during two remotely-delivered weight loss interventions. *International Journal Of Medical Informatics*, *83*(5), 313–319. Exclusion Reason: 7

Pekmezi, D., Ainsworth, C., Holly, T., Williams, V., Benitez, T., Wang, K., … Demark-Wahnefried, W. (2017). Rationale, design, and baseline findings from a pilot randomized trial of an IVR-Supported physical activity intervention for cancer prevention in the Deep South: The DIAL study. *Contemporary Clinical Trials Communications*, *8*(October), 218–226. Exclusion Reason: 3

Neubeck, L., Coorey, G., Peiris, D., Mulley, J., Heeley, E., Hersch, F., & Redfern, J. (2016). Development of an integrated e-health tool for people with, or at high risk of, cardiovascular disease: The Consumer Navigation of Electronic Cardiovascular Tools (CONNECT) web application. *International Journal of Medical Informatics*, *96*, 24–37. Exclusion Reason: 1

Simons, D., De Bourdeaudhuij, I., Clarys, P., De Cocker, K., Vandelanotte, C., & Deforche, B. (2018). A Smartphone App to Promote an Active Lifestyle in Lower-Educated Working Young Adults: Development, Usability, Acceptability, and Feasibility Study. *JMIR MHEALTH AND UHEALTH*, *6*(2), e44. Exclusion Reason: 8

Read, E. (2014). Feasibility of the diabetes and technology for increased activity (DaTA) study: A pilot intervention in high-risk rural adults. *Journal of Physical Activity and Health*, *11*(1), 118–126. Exclusion Reason: 7

Coppini, G., Zuccaia, V. C., De Maria, R., Nazare, J. A., Morales, M. A., & Colantonio, S. (2017). User acceptance of self-monitoring technology to prevent cardio-metabolic diseases: The Wize Mirror. *International Conference on Wireless and Mobile Computing, Networking and Communications*. Exclusion Reason: 1

Mayette, G. G. (2016). *Evaluating a Novel Device for Calorie Reduction : The Bite Counter Study*. *University of Rhode Island*. University of Rhode Island. Exclusion Reason: 1

Jeyabalan, K. (2017). Home healthcare and remote patient monitoring. In *Internet of Things and Data Analytics Handbook* (pp. 675–682). University of Utah, Salt Lake City, UT, United States: wiley. Exclusion Reason: 1

Massone, C., Hofmann-Wellenhof, R., Ahlgrimm-Siess, V., Gabler, G., Ebner, C., & Soyer, H. P. (2007). Melanoma screening with cellular phones. *Plos One*, *2*(5), e483–e483. Exclusion Reason: 1

Allen, J. D., Othus, M. K. D., Hart Jr., A., Tom, L., Li, Y., Berry, D., & Bowen, D. (2010). A randomized trial of a computer-tailored decision aid to improve prostate cancer screening decisions: results from the Take the Wheel trial. *Cancer Epidemiology, Biomarkers & Prevention*, *19*(9), 2172–2186. Exclusion Reason: 1

Kharrazi, H., & Vincz, L. (2011). Increasing Physical Activity by Implementing a Behavioral Change Intervention Using Pervasive Personal Health Record System: An Exploratory Study. In C. Stephanidis (Ed.), *Universal Access in Human-Computer Interaction: Applications and Services,* (Vol. 6768, pp. 366–375). Exclusion Reason: 3

Dunn, J. T. (2017). *Data integration for health and stress monitoring: Biological metabolites, wearables data, and self-reporting*. ProQuest Information & Learning, US. Exclusion Reason: 1

Soureti, A., Murray, P., Cobain, M., Chinapaw, M., van Mechelen, W., & Hurling, R. (2011). Exploratory study of web-based planning and mobile text reminders in an overweight population. *Journal Of Medical Internet Research*, *13*(4), e118–e118. Exclusion Reason: 1

Linas, B. S., Latkin, C., Genz, A., Westergaard, R. P., Chang, L. W., Bollinger, R. C., & Kirk, G. D. (2015). Utilizing mHealth methods to identify patterns of high risk illicit drug use. *Drug and Alcohol Dependence*, *151*, 250–257. Exclusion Reason: 2

Cohn, W. F., Ropka, M. E., Pelletier, S. L., Barrett, J. R., Kinzie, M. B., Harrison, M. B., … Knaus, W. A. (2010). Health Heritage© a web-based tool for the collection and assessment of family health history: initial user experience and analytic validity. *Public Health Genomics*, *13*(7–8), 477–491. Exclusion Reason: 1

Bewick, B. M., Trusler, K., Mulhern, B., Barkham, M., & Hill, A. J. (2008). The feasibility and effectiveness of a web-based personalised feedback and social norms alcohol intervention in UK university students: A randomised control trial. *Addictive Behaviors*, *33*(9), 1192–1198. Exclusion Reason: 1

Brown, J., Michie, S., Geraghty, A. W. A., Miller, S., Yardley, L., Gardner, B., … West, R. (2012). A pilot study of StopAdvisor: a theory-based interactive internet-based smoking cessation intervention aimed across the social spectrum. *Addictive Behaviors*, *37*(12), 1365–1370. Exclusion Reason: 1

Ganapathy, R., Grewal, A., & Castleman, J. S. (2016). Remote monitoring of blood pressure to reduce the risk of preeclampsia related complications with an innovative use of mobile technology. *Pregnancy Hypertension*, *6*(4), 263–265. Exclusion Reason: 2

Wilkinson, L., Pacanowski, C., & Levitsky, D. (2016). Self-weighing and visual feedback has long term effects on weight gain prevention in adults. *FASEB Journal*, *30*. Exclusion Reason: 1

Allen, J. D., Mohllajee, A. P., Shelton, R. C., Drake, B. F., & Mars, D. R. (2009). A computer-tailored intervention to promote informed decision making for prostate cancer screening among African American men. *American Journal Of Men's Health*, *3*(4), 340–351. Exclusion Reason: 1

Davis, S., Abidi, S., & Cox, J. (2008). Generating personalised cardiovascular risk management educational interventions linking SCORE and behaviour change. *Journal on Information Technology in Healthcare*, *6*(1), 73–82. Exclusion Reason: 3

Elfeddali, I., Bolman, C., & de Vries, H. (2013). Situational and Affective Risk Situations of Relapse and the Quality of Implementation Intentions in an E-Health Smoking Relapse Prevention Programme. *Substance Use & Misuse*, *48*(8), 635–644. **Exclusion Reason: 1**

Kukafka, R., Jeong, I. C., Finkelstein, J., & Ieee. (2015). Introducing Decision Support for Smart Mobile Health Behavior Change Applications. In *2015 International Conference on Big Data and Smart Computing* (pp. 75–78). **Exclusion Reason: 1**

BinDhim, N. F., Shaman, A. M., Trevena, L., Basyouni, M. H., Pont, L. G., & Alhawassi, T. M. (2015). Depression screening via a smartphone app: cross-country user characteristics and feasibility. *Journal Of The American Medical Informatics Association: JAMIA*, *22*(1), 29–34. **Exclusion Reason: 1**

Wanner, M., Martin-Diener, E., Braun-Fahrländer, C., Bauer, G., & Martin, B. W. (2009). Effectiveness of active-online, an individually tailored physical activity intervention, in a real-life setting: randomized controlled trial. *Journal Of Medical Internet Research*, *11*(3), e23–e23. **Exclusion Reason: 1**

Voncken-Brewster, V., Tange, H., de Vries, H., Nagykaldi, Z., Winkens, B., & van der Weijden, T. (2015). A randomized controlled trial evaluating the effectiveness of a web-based, computer-tailored self-management intervention for people with or at risk for COPD. *International Journal Of Chronic Obstructive Pulmonary Disease*, *10*, 1061–1073. **Exclusion Reason: 1**

Nagykaldi, Z. J., Voncken-Brewster, V., Aspy, C. B., & Mold, J. W. (2013). Novel computerized health risk appraisal may improve longitudinal health and wellness in primary care: a pilot study. *Applied Clinical Informatics*, *4*(1), 75–87. **Exclusion Reason: 1**

Wu, H. (2013). *Exploring healthcare consumer acceptance of personal health information management technology through personal health record systems*. ProQuest Information & Learning, Baltimore County. **Exclusion Reason: 1**

Bashford, G. R., Burnfield, J. M., & Pérez, L. C. (2013). Physical activity discrimination improvement using accelerometers and wireless sensor network localization. *Biomedical Sciences Instrumentation*, *49*, 243–250. **Exclusion Reason: 1**

Partridge, S. R., Allman-Farinelli, M., McGeechan, K., Balestracci, K., Wong, A. T. Y., Hebden, L., … Phongsavan, P. (2016). Process evaluation of TXT2BFiT: a multi-component mHealth randomised controlled trial to prevent weight gain in young adults. *The International Journal Of Behavioral Nutrition And Physical Activity*, *13*, 7. **Exclusion Reason: 1**

Botts, N. E., Horan, T. A., & Thoms, B. P. (2011). HealthATM: Personal health cyberinfrastructure for underserved populations. *American Journal of Preventive Medicine*, *40*(5 SUPPL. 2), S115–S122. **Exclusion Reason: 1**

Wolin, K. Y., Steinberg, D. M., Lane, I. B., Askew, S., Greaney, M. L., Colditz, G. A., & Bennett, G. G. (2015). Engagement with eHealth Self-Monitoring in a Primary Care-Based Weight Management Intervention. *Plos One*, *10*(10), e0140455–e0140455. Exclusion Reason: 1

Spook, J. E., Paulussen, T., Kok, G., & Van Empelen, P. (2013). Monitoring dietary intake and physical activity electronically: feasibility, usability, and ecological validity of a mobile-based Ecological Momentary Assessment tool. *Journal Of Medical Internet Research*, *15*(9), e214–e214. Exclusion Reason: 1

Saitz, R., Helmuth, E. D., Aromaa, S. E., Guard, A., Belanger, M., & Rosenbloom, D. L. (2004). Web-based screening and brief intervention for the spectrum of alcohol problems. *Preventive Medicine*, *39*(5), 969–975. Exclusion Reason: 1

Martin, C. K., Gilmore, L. A., Apolzan, J. W., Myers, C. A., Thomas, D. M., & Redman, L. M. (2016). Smartloss: A Personalized Mobile Health Intervention for Weight Management and Health Promotion. *JMIR Mhealth And Uhealth*, *4*(1), e18–e18. Exclusion Reason: 3

Chih, M.-Y., Patton, T., McTavish, F. M., Isham, A. J., Judkins-Fisher, C. L., Atwood, A. K., & Gustafson, D. H. (2014). Predictive modeling of addiction lapses in a mobile health application. *Journal Of Substance Abuse Treatment*, *46*(1), 29–35. Exclusion Reason: 2

Michaelides, A., Raby, C., Wood, M., Farr, K., & Toro-Ramos, T. (2016). Weight loss efficacy of a novel mobile Diabetes Prevention Program delivery platform with human coaching. *BMJ Open Diabetes Research & Care*, *4*(1), e000264. Exclusion Reason: 2

Hensel, D. J., Fortenberry, J. D., Harezlak, J., & Craig, D. (2012). The feasibility of cell phone based electronic diaries for STI/HIV research. *BMC Medical Research Methodology*, *12*, 75. Exclusion Reason: 2

Batch, B. C., Tyson, C., Bagwell, J., Corsino, L., Intille, S., Lin, P. H., … Svetkey, L. P. (2014). Weight loss intervention for young adults using mobile technology: Design and rationale of a randomized controlled trial - Cell Phone Intervention for You (CITY). *Contemporary Clinical Trials*, *37*(2), 333–341. Exclusion Reason: 3

Tucker, J. A., Foushee, H. R., Black, B. C., & Roth, D. L. (2007). Agreement between prospective interactive voice response self-monitoring and structured retrospective reports of drinking and contextual variables during natural resolution attempts. *Journal of Studies on Alcohol and Drugs*, *68*(4), 538–542. Exclusion Reason: 1

Calhoun, P. S., Datta, S., Olsen, M., Smith, V. A., Moore, S. D., Hair, L. P., … Bastian, L. A. (2016). Comparative effectiveness of an Internet-based smoking cessation intervention versus clinic-based specialty care for veterans. *Journal of Substance Abuse Treatment*, *69*, 19–27. Exclusion Reason: 1

Bonilla, C., Brauer, P., Royall, D., Keller, H., Hanning, R. M., & Dicenso, A. (2015). Use of electronic dietary assessment tools in primary care: An interdisciplinary perspective eHealth/ telehealth/ mobile health systems. *BMC Medical Informatics and Decision Making*, *15*(1). Exclusion Reason: 3

Wright, A., Poon, E. G., Wald, J., Schnipper, J. L., Grant, R., Gandhi, T. K., … Middleton, B. (2008). Effectiveness of health maintenance reminders provided directly to patients. In *AMIA ... Annual Symposium proceedings / AMIA Symposium. AMIA Symposium* (p. 1183). A. Wright, Partners HealthCare System, Brigham and Women’s Hospital, Massachusetts General Hospital, Harvard Medical School, Boston, MA, USA. Exclusion Reason: 1

Munir, F., Biddle, S. J. H., Davies, M. J., Dunstan, D., Esliger, D., Gray, L. J., … Edwardson, C. L. (2018). Stand More AT Work (SMArT Work): using the behaviour change wheel to develop an intervention to reduce sitting time in the workplace. *BMC Public Health*, *18*(1), 319. Exclusion Reason: 1

Lanpher, M. G., Askew, S., & Bennett, G. G. (2016). Health Literacy and Weight Change in a Digital Health Intervention for Women: A Randomized Controlled Trial in Primary Care Practice. *Journal Of Health Communication*, *21 Suppl 1*, 34–42. Exclusion Reason: 1

Buckingham, C. D., Adams, A., Vail, L., Kumar, A., Ahmed, A., Whelan, A., & Karasouli, E. (2015). Integrating service user and practitioner expertise within a web-based system for collaborative mental-health risk and safety management. *Patient Education And Counseling*, *98*(10), 1189–1196. Exclusion Reason: 3

Smith, S. G., O’Conor, R., Aitken, W., Curtis, L. M., Wolf, M. S., & Goel, M. S. (2015). Disparities in registration and use of an online patient portal among older adults: findings from the LitCog cohort. *Journal of the American Medical Informatics Association*, *22*(4), 888–895. Exclusion Reason: 3

Shrier, L. A., Rhoads, A., Burke, P., Walls, C., & Blood, E. A. (2014). Real-time, contextual intervention using mobile technology to reduce marijuana use among youth: a pilot study. *Addictive Behaviors*, *39*(1), 173–180. Exclusion Reason: 1

Omar, A. (2009). Pitfalls and successes of a web-based wellness program. In A. A. Lazakidou, K. M. Siassiakos, A. A. Lazakidou, & K. M. Siassiakos (Eds.), *Handbook of research on distributed medical informatics and e-health.* (pp. 137–151). Hershey, PA, US: Medical Information Science Reference/IGI Global. Exclusion Reason: 1

Scanlan, B., McTigue, K. M., Wang, L., Winger, D., Conroy, M. B., B., S., … M.B., C. (2015). Association of eating habits with weight loss and diet self-monitoring in an online weight loss trial: Results from the Ocelot PC-Study. *Journal of General*, *30*(2), S105. Exclusion Reason: 1

Steinberg, D. M., Levine, E. L., Lane, I., Askew, S., Foley, P. B., Puleo, E., & Bennett, G. G. (2014). Adherence to self-monitoring via interactive voice response technology in an eHealth intervention targeting weight gain prevention among Black women: randomized controlled trial. *Journal Of Medical Internet Research*, *16*(4), e114–e114. Exclusion Reason: 1

Esch, S., Knebel, U., Leimeister, J. M., & Krcmar, H. (2009). Design, implementation and test of an IT architecture for a mobile health coach: The personal health manager as an example. In A. Rashid, T. Eymann, & J. M. Leimeister (Eds.), *Mobiles Computing in der*

Medizin - Proceedings zum 9. Workshop der GI- und GMDS-Arbeitsgruppe Mobile Informationstechnologie in der Medizin, MoCoMed 2009 - 9th Workshop of the GI- and GMDS Working Group on Mobile Information Technology in Medicine, MoCoMe (pp. 12–17). Lehrstuhl für Wirtschaftsinformatik, Technische Universität München, Exclusion Reason: 3

Omar, A., Wahlqvist, M. L., Kouris-Blazos, A., & Vicziany, M. (2005). Wellness management through Web-based programmes. *Journal Of Telemedicine And Telecare*, *11 Suppl 1*, 8–11. Exclusion Reason: 1

Elfeddali, I., Bolman, C., Candel, M. J. J. M., Wiers, R. W., & de Vries, H. (2012). Preventing smoking relapse via Web-based computer-tailored feedback: a randomized controlled trial. *Journal Of Medical Internet Research*, *14*(4), e109–e109. Exclusion Reason: 1

McDaniel, A. M., Benson, P. L., Roesener, G. H., & Martindale, J. (2005). An integrated computer-based system to support nicotine dependence treatment in primary care. *Nicotine and Tobacco Research*, *7*(SUPPL. 1), S57–S66. Exclusion Reason: 1

Sequist, T. D., Zaslavsky, A. M., Colditz, G. A., & Ayanian, J. Z. (2011). Electronic patient messages to promote colorectal cancer screening: a randomized controlled trial. *Archives Of Internal Medicine*, *171*(7), 636–641. Exclusion Reason: 1

Verdezoto, N., & Grönvall, E. (2016). On preventive blood pressure self-monitoring at home. *Cognition, Technology and Work*, *18*(2), 267–285. Shieh, C., Yang, Z., Haas, D. M., & Carpenter, J. S. (2017). Feasibility and Potential Benefits of a Self-Monitoring Enhanced Lifestyle Intervention to Prevent Excessive Gestational Weight Gain in Women Who Are Overweight or Obese. *Journal of Obstetric, Gynecologic, and Neonatal Nursing : JOGNN*, *46*(2), 182–196. Exclusion Reason: 1

Rose, G. L., Badger, G. J., Skelly, J. M., MacLean, C. D., Ferraro, T. A., & Helzer, J. E. (2017). A randomized controlled trial of brief intervention by interactive voice response. *Alcohol and Alcoholism*, *52*(3), 335–343. Exclusion Reason: 1

Kelders, S. M., Van Gemert-Pijnen, J. E. W. C., Werkman, A., Nijland, N., & Seydel, E. R. (2011). Effectiveness of a Web-based intervention aimed at healthy dietary and physical activity behavior: a randomized controlled trial about users and usage. *Journal Of Medical Internet Research*, *13*(2), e32–e32. Exclusion Reason: 1

Li, Y., Wu, C., Guo, L., Lee, C.-H., & Guo, Y. (2014). Wiki-health: A big data platform for health sensor data management. In *Cloud Computing Applications for Quality Health Care Delivery* (pp. 59–77). Department of Computing, Imperial College London, United Kingdom School of Computing, Engineering, and Computer Science, University of Central Lancashire, United Kingdom Imperial College London, United Kingdom: IGI Global. Exclusion Reason: 1

Basch, E., & Goldfard, S. (2009). Electronic patient-reported outcomes for collecting sensitive information from patients. *Journal of Supportive Oncology*, *7*(3), 98–99. Exclusion Reason: 1

Smallwood, A. J., Schapira, M. M., Fedders, M., & Neuner, J. M. (2017). A pilot randomized controlled trial of a decision aid with tailored fracture risk tool delivered via a patient portal. *Osteoporosis International*, *28*(2), 567–576. Exclusion Reason: 1

Levenson, J. C., Miller, E., Hafer, B., Reidell, M. F., Buysse, D. J., & Franzen, P. L. (2016). Pilot Study of a Sleep Health Promotion Program for College Students. *Sleep Health*, *2*(2), 167–174. Exclusion Reason: 1

Hutchesson, M. J., Tan, C. Y., Morgan, P., Callister, R., & Collins, C. (2016). Enhancement of Self-Monitoring in a Web-Based Weight Loss Program by Extra Individualized Feedback and Reminders: Randomized Trial. *Journal of Medical Internet Research*, *18*(4). Exclusion Reason: 2

Liu, S., Hodgson, C., Zbib, A. M., Payne, A. Y. M., & Nolan, R. P. (2014). The effectiveness of loyalty rewards to promote the use of an internet-based heart health program. *Journal of Medical Internet Research*, *16*(7), 6–13. Exclusion Reason: 1

Savic, M., Barker, S. F., Hunter, B., & Lubman, D. I. (2016). 'Holy shit, didn't realise my drinking was high risk': an analysis of the way risk is enacted through an online alcohol and drug screening intervention. *Health, Risk and Society*, *17*(7–8), 565–591. Exclusion Reason: 1

Wald, J. S., Middleton, B., Bloom, A., Walmsley, D., Gleason, M., Nelson, E., … Bates, D. W. (2004). A patient-controlled journal for an electronic medical record: issues and challenges. *Medinfo*, *11*(Pt 2), 1166–1170. Exclusion Reason: 3

Rubinstein, A., Miranda, J. J., Beratarrechea, A., Diez-Canseco, F., Kanter, R., Gutierrez, L., … Ramirez-Zea, M. (2016). Effectiveness of an mHealth intervention to improve the cardiometabolic profile of people with prehypertension in low-resource urban settings in Latin America: a randomised controlled trial. *The Lancet. Diabetes & Endocrinology*, *4*(1), 52–63. Exclusion Reason: 1

Weingart, S. N., Carbo, A., Tess, A., Chiappetta, L., Tutkus, S., Morway, L., … Bates, D. W. (2013). Using a patient internet portal to prevent adverse drug events: a randomized, controlled trial. *Journal Of Patient Safety*, *9*(3), 169–175. Exclusion Reason: 1

Akram, A., Dunleavy, G., Soljak, M., & Car, J. (2017). Beyond health apps, utilize patient-generated data. In R. Damasevieius & V. Mikasyte (Eds.), *23rd International Conference on Information and Software Technologies, ICIST 2017* (Vol. 756, pp. 65–76). Centre for Population Health Sciences (CePHaS), LKC Medicine, Nanyang Technological University, Singapore, Singapore Global eHealth Unit, Department of Primary Care and Public Health, School of Public Health, Imperial College, London, United Kingdom: Springer Verlag. Exclusion Reason: 2

Lau, A. Y. S., Sintchenko, V., Crimmins, J., Magrabi, F., Gallego, B., & Coiera, E. (2012). Impact of a web-based personally controlled health management system on influenza vaccination and health services utilization rates: a randomized controlled trial. *Journal Of The American Medical Informatics Association*, *19*(5), 719–727. Exclusion Reason: 1

Ledford, C. J. W., Canzona, M. R., Cafferty, L. A., & Hodge, J. A. (2016). Mobile application as a prenatal education and engagement tool: A randomized controlled pilot. *Patient Education and Counseling*, *99*(4), 578–582. **Exclusion Reason: 1**

Storm, V., Dörenkämper, J., Reinwand, D. A., Wienert, J., De Vries, H., & Lippke, S. (2016). Effectiveness of a web-based computer-tailored multiple-lifestyle intervention for people interested in reducing their cardiovascular risk:A randomized controlled trial. *Journal of Medical Internet Research*, *18*(4), 35–49. **Exclusion Reason: 1**

Babiloni, C., Guarini, A., Lizio, R., Cordone, S., Bonaduce, R., Armenise, V., … Aldera, C. (2016). Prevention of mental disorders in seniors at risk of Alzheimer's disease in the smart health project: The smartaging platform. *Alzheimer's and Dementia*, *12*(7), P592–P593. **Exclusion Reason: 1**

Wagner, P. J., Howard, S. M., Bentley, D. R., Seol, Y.-H., & Sodomka, P. (2010). Incorporating patient perspectives into the personal health record: implications for care and caring. *Perspectives In Health Information Management*, *7*, 1e–1e. **Exclusion Reason: 2**

Wangberg, S. C., Nilsen, O., Antypas, K., & Gram, I. T. (2011). Effect of tailoring in an internet-based intervention for smoking cessation: randomized controlled trial. *Journal Of Medical Internet Research*, *13*(4), e121–e121. **Exclusion Reason: 1**

Melchart, D., Doerfler, W., Eustachi, A., Wellenhofer-Li, Y., & Weidenhammer, W. (2015). The talent study: a multicentre randomized controlled trial assessing the impact of a "tailored lifestyle self-management intervention" (talent) on weight reduction. *BMC Obesity*, *2*, 38. **Exclusion Reason: 1**

Bewick, B. M., West, R., Gill, J., O'May, F., Mulhern, B., Barkham, M., & Hill, A. J. (2010). Providing web-based feedback and social norms information to reduce student alcohol intake: a multisite investigation. *Journal Of Medical Internet Research*, *12*(5), e59–e59. **Exclusion Reason: 1**

Ho, C. S., & Lin, P. I. (2013). Cloud-based Bayesian inference for online people health status assessment. In Y. H. Kim & P. Yarlagadda (Eds.), *Applied Mechanics and Materials* (Vol. 303–306, p. 2231–+). Ho, CS (reprint author), 58,Sec 2,Sih Chuan Rd, New Taipei City 22061, Taiwan. FL007@mail.oit.edu.tw; FL002@mail.vnu.edu.tw: Trans Tech Publications Ltd. https://doi.org/10.4028/www.scientific.net/AMM.303-306.2231 **Exclusion Reason: 3**

García-Gómez, J. M., de la Torre-Díez, I., Vicente, J., Robles, M., López-Coronado, M., & Rodrigues, J. J. (2014). Analysis of mobile health applications for a broad spectrum of consumers: a user experience approach. *Health Informatics Journal*, *20*(1), 74–84. **Exclusion Reason: 3**

Aschettino, L., Baldwin, K., Friedman, B., Grady, R., Grebner, L., Hennings, M. E., … Washington, L. (2015). Including Patient-Generated Health Data in Electronic Health Records. *Journal Of AHIMA*, *86*(2), 54–57. **Exclusion Reason: 1**

Young-Mason, J. (2013). Information and knowledge is power: the online personal health record. *Clinical Nurse Specialist CNS*, *27*(2), 105–106. Exclusion Reason: 1

Forjuoh, S. N., Ory, M. G., Wang, S. J., des Bordes, J. K. A., & Hong, Y. (2014). Using the iPod Touch for Patient Health Behavior Assessment and Health Promotion in Primary Care. *Jmir Mhealth and Uhealth*, *2*(1), 10. Exclusion Reason: 1

Stuckey, M., Gill, D., Shapiro, S., Sabourin, K., & Petrella, R. (2013). Does prescriptive exercise with mobile health tracking improve self-efficacy and health status in individuals with metabolic syndrome? *Circulation*. Exclusion Reason: 2

Sinadinovic, K., Berman, A. H., Hasson, D., & Wennberg, P. (2010). Internet-based assessment and self-monitoring of problematic alcohol and drug use. *Addictive Behaviors*, *35*(5), 464–470. Exclusion Reason: 1

Greaney, M. L., Sprunck-Harrild, K., Bennett, G. G., Puleo, E., Haines, J., Viswanath, K. V., & Emmons, K. M. (2012). Use of Email and Telephone Prompts to Increase Self-Monitoring in a Web-Based Intervention: Randomized Controlled Trial. *Journal of Medical Internet Research*, *14*(4), 76–86. Exclusion Reason: 2

Svetlak, M., & Kukleta, M. (2006). Smokers' self-monitoring scale: A pilot study of possibilities of a graphic form. *Homeostasis in Health and Disease*, *44*(1–2), 75–77. Exclusion Reason: 1

Plaete, J., De Bourdeaudhuij, I., Verloigne, M., & Crombez, G. (2015). Acceptability, feasibility and effectiveness of an eHealth behaviour intervention using self-regulation: "MyPlan." *Patient Education and Counseling*, *98*(12), 1617–1624. Exclusion Reason: 1

Tomko, C., Davis, K. M., Luta, G., Krist, A. H., Woolf, S. H., & Taylor, K. L. (2015). A comparison of web-based versus print-based decision AIDS for prostate cancer screening: participants' evaluation and utilization. *Journal Of General Internal Medicine*, *30*(1), 33–42. Exclusion Reason: 1

Charlon, Y., Campo, E., & Brulin, D. (2018). Design and evaluation of a smart insole: Application for continuous monitoring of frail people at home. *Expert Systems with Applications*, *95*, 57–71. Exclusion Reason: 7

Zarei, R., & Kuo, A. (2017). Design and Development of a Web-Based Self-Monitoring System to Support Wellness Coaching. In A. Lau, F and BartleClar, J and Bliss, G and Borycki, E and Courtney, K and Kuo (Ed.), *ITCH* (Vol. 234, pp. 401–406). Exclusion Reason: 3

Willcox, J. C., Wilkinson, S. A., Lappas, M., Ball, K., Crawford, D., McCarthy, E. A., … Campbell, K. J. (2017). A mobile health intervention promoting healthy gestational weight gain for women entering pregnancy at a high body mass index: the txt4two pilot randomised controlled trial. *BJOG: An International Journal Of Obstetrics And Gynaecology*, *124*(11), 1718–1728. Exclusion Reason: 1

Sheehan, B., & Lucero, R. J. (2015). Initial Usability and Feasibility Evaluation of a Personal Health Record-Based Self-Management System for Older Adults. *EGEMS (Washington, DC)*, *3*(2), 1152. Exclusion Reason: 3

Poppe, L., Van Der Mispel, C., De Bourdeaudhuij, I., Verloigne, M., Shadid, S., & Crombez, G. (2017). Users' thoughts and opinions about a self-regulation-based eHealth intervention targeting physical activity and the intake of fruit and vegetables: A qualitative study. *PLoS ONE*, *12*(12). Exclusion Reason: 1

Bonacina, S., Privitera, Y. A., Marsilio, S., Montin, E., Passarelli, F., Masseroli, M., & Pinciroli, F. (2009). A web-based tool for cooperating behaviors in eating and physical activity control. *Annual Review of CyberTherapy and Telemedicine*, *7*, 155–159. Exclusion Reason: 3

Partridge, S. R., McGeechan, K., Bauman, A., Phongsavan, P., & Allman-Farinelli, M. (2016). Improved eating behaviours mediate weight gain prevention of young adults: moderation and mediation results of a randomised controlled trial of TXT2BFiT, mHealth program. *The International Journal Of Behavioral Nutrition And Physical Activity*, *13*, 44. Exclusion Reason: 1

Spielberg, F., Levy, V., Lensing, S., Chattopadhyay, I., Venkatasubramanian, L., Acevedo, N., … Gaydos, C. A. (2014). Fully integrated e-services for prevention, diagnosis, and treatment of sexually transmitted infections: results of a 4-county study in California. *American Journal Of Public Health*, *104*(12), 2313–2320. Exclusion Reason: 1

Takata, Y., Ou, O., Nishida, H., & Sakagami, K. (2002). Impact of self-monitoring of blood glucose on the lifestyles of subjects with fasting hyperglycemia: A randomized controlled trial. *Journal of Occupational Health*, *44*(1), 28–33. Exclusion Reason: 1

Burke, L. E., Sereika, S., Choo, J., Warziski, M., Music, E., Styn, M., … Stone, A. (2006). Ancillary study to the PREFER trial: a descriptive study of participants' patterns of self-monitoring--rationale, design and preliminary experiences. *Contemporary Clinical Trials*, *27*(1), 23–33. Exclusion Reason: 1

Gay, V., & Leijdekkers, P. (2015). Bringing Health and Fitness Data Together for Connected Health Care: Mobile Apps as Enablers of Interoperability. *Journal Of Medical Internet Research*, *17*(11), e260–e260. Exclusion Reason: 3

Mason, D., Gilbert, H., & Sutton, S. (2012). Effectiveness of web-based tailored smoking cessation advice reports (iQuit): a randomized trial. *Addiction (Abingdon, England)*, *107*(12), 2183–2190. Exclusion Reason: 1

Mañanes, G., & Vallejo, M. A. (2014). Usage and effectiveness of a fully automated, open-access, Spanish Web-based smoking cessation program: randomized controlled trial. *Journal Of Medical Internet Research*, *16*(4), e111–e111. Exclusion Reason: 1

van der Krieke, L., Blaauw, F. J., Emerencia, A. C., Schenk, H. M., Slaets, J. P. J., Bos, E. H., … Jeronimus, B. F. (2017). Temporal Dynamics of Health and Well-Being: A Crowdsourcing

Approach to Momentary Assessments and Automated Generation of Personalized Feedback. *Psychosomatic Medicine*, *79*(2), 213–223. Exclusion Reason: 1

Forster, H., Walsh, M., Drevon, C., Manios, Y., Traczyk, I., Martinez, J., … Brennan, L. (2015). Development and automation of a dietary feedback system for the delivery of personalised dietary advice. In *Proceedings of the nutrition society. Conference: irish section meeting - nutrition at key life stages: new findings, new approaches. Ireland* (Vol. 74). Exclusion Reason: 1

Baldacchino, A., Crocamo, C., Humphris, G., Neufeind, J., Frisher, M., Scherbaum, N., & Carrà, G. (2016). Decision support in addiction: The development of an e-health tool to assess and prevent risk of fatal overdose. The ORION Project. *Computer Methods And Programs In Biomedicine*, *133*, 207–216. Exclusion Reason: 1

Jones, J. B., Shah, N. R., Bruce, C. A., & Stewart, W. F. (2011). Meaningful use in practice using patient-specific risk in an electronic health record for shared decision making. *American Journal Of Preventive Medicine*, *40*(5 Suppl 2), S179–S186. Exclusion Reason: 1

Pittman, J. O. E., Floto, E., Lindamer, L., Baker, D. G., Lohr, J. B., & Afari, N. (2017). VA eScreening program: Technology to improve care for post-9/11 veterans. *Psychological Services*, *14*(1), 23–33. Exclusion Reason: 1

Whelan, M. E., Kingsnorth, A. P., Orme, M. W., Sherar, L. B., & Esliger, D. W. (2017). Sensing interstitial glucose to nudge active lifestyles (SIGNAL): Feasibility of combining novel self-monitoring technologies for persuasive behaviour change. *BMJ Open*, *7*(10). Exclusion Reason: 3

Kukafka, R., Jeong, I. cheol, & Finkelstein, J. (2015). Optimizing Decision Support for Tailored Health Behavior Change Applications. *Studies In Health Technology And Informatics*, *216*, 108–112. Exclusion Reason: 1

Stevens, V. J., Funk, K. L., Brantley, P. J., Erlinger, T. P., Myers, V. H., Champagne, C. M., … Hollis, J. F. (2008). Design and implementation of an interactive website to support long-term maintenance of weight loss. *Journal of Medical Internet Research*, *10*(1), e1. Exclusion Reason: 2

Brendryen, H., Drozd, F., & Kraft, P. (2008). A digital smoking cessation program delivered through internet and cell phone without nicotine replacement (happy ending): randomized controlled trial. *Journal Of Medical Internet Research*, *10*(5), e51–e51. Exclusion Reason: 1

Steinhubl, S., Waalen, J., Edwards, A., Mehta, R., Ariniello, L., Ebner, G., … Topol, E. (2017). A nationwide trial of screening for undiagnosed atrial fibrillation in an at-risk population using a self-applied ECG recording patch sensor: results of the Randomized Component of the mHealth Screening to Prevent Strokes (mSToPS) trial. *Circulation. Conference: Resuscitation Science Symposium, Ress 2017. United States*. Exclusion Reason: 1

Nahm, E. S., Diblasi, C., Gonzales, E., Silver, K., Zhu, S., Sagherian, K., & Kongs, K. (2017). Patient-Centered Personal Health Record and Portal Implementation Toolkit for Ambulatory Clinics: A Feasibility Study. *Computers, Informatics, Nursing : CIN*, *35*(4), 176–185. Exclusion Reason: 2

van Osch, M., Rovekamp, A. J. M., Bergman-Agteres, S. N., Wijsman, L. W., Ooms, S. J., Mooijaart, S. P., & Vermeulen, J. (2015). User preferences and usability of iVitality: optimizing an innovative online research platform for home-based health monitoring. *Patient Preference and Adherence*, *9*, 857–867. Exclusion Reason: 2

Schleyer, T., King, Z., & Miled, Z. B. (2016). A Novel Conceptual Architecture for Person-Centered Health Records. In *AMIA ... Annual Symposium proceedings. AMIA Symposium* (Vol. 2016, pp. 1090–1099). Exclusion Reason: 3

Schulz, D. N., Candel, M. J., Kremers, S. P., Reinwand, D. A., Jander, A., & de Vries, H. (2013). Effects of a Web-based tailored intervention to reduce alcohol consumption in adults: randomized controlled trial. *Journal of Medical Internet Research*, *15*(9), e206. Exclusion Reason: 1

Kuvaja-Köllner, V., Steffansson, M., & Kettunen, A. (2013). Information and communications technology-supported diabetes prevention and self-care management: experiences from the EMOTIONAAL project in Finland. *Journal Of Diabetes Science And Technology*, *7*(1), 287–288. Exclusion Reason: 1

Boateng, G., Batsis, J. A., Halter, R., Kotz, D., & Ieee. (2017). ActivityAware: An App for Real-Time Daily Activity Level Monitoring on the Amulet Wrist-Worn Device. In *2017 Ieee International Conference on Pervasive Computing and Communications Workshops*. [Boateng, George; Batsis, John A.; Halter, Ryan; Kotz, David] Dartmouth Coll, Hanover, NH 03755 USA. Boateng, G (reprint author), Dartmouth Coll, Hanover, NH 03755 USA.: IEEE. Exclusion Reason: 1

Burke, P., & Shrier, L. (2016). Use of Mobile Technology to Reduce Marijuana Use Among Adolescents and Young Adults...28th Annual Scientific Session, June 2-6, 2017, Baltimore, Maryland. *Nursing Research*, *65*(2), E71–E71. Exclusion Reason: 1

Lenert, L., Munoz, R. F., Stoddard, J., Delucchi, K., Bansod, A., Skoczen, S., & Perez-Stable, E. J. (2003). Design and pilot evaluation of an Internet smoking cessation program. *Journal of the American Medical Informatics Association*, *10*(1), 16–20. Exclusion Reason: 1

Scanlan, B., Conroy, M. B., Tudorascu, D. L., Karpov, I., Hess, R., G., F., … McTigue, K. M. (2014). Association of adherence measures with physical activity outcomes in an online weight loss trial: Results from the ocelot study. *Journal of General Internal Medicine*, *29*, S32–S33. Exclusion Reason: 1

Carrà, G., Crocamo, C., Humphris, G., Tabacchi, T., Bartoli, F., Neufeind, J., … Baldacchino, A. (2017). Engagement in the Overdose RIsk InfOrmatioN (ORION) e-Health Tool for Opioid Overdose Prevention and Self-Efficacy: A Preliminary Study. *Cyberpsychology, Behavior And Social Networking*, *20*(12), 762–768. Exclusion Reason: 1

Bourgeois, F. T., Simons, W. W., Olson, K., Brownstein, J. S., & Mandl, K. D. (2008). Evaluation of influenza prevention in the workplace using a personally controlled health record: randomized controlled trial. *Journal Of Medical Internet Research*, *10*(1), e5–e5. Exclusion Reason: 1

Rossen, J., Yngve, A., Hagstromer, M., Brismar, K., Ainsworth, B. E., Iskull, C., … Johansson, U.-B. (2015). Physical activity promotion in the primary care setting in pre- and type 2 diabetes - the Sophia step study, an RCT. *BMC Public Health*, *15*, 647. Exclusion Reason: 2

Gillaspy, S. R., Leffingwell, T., Mignogna, M., Mignogna, J., Bright, B., & Fedele, D. (2013). Testing of a web-based program to facilitate parental smoking cessation readiness in primary care. *Journal Of Primary Care & Community Health*, *4*(1), 2–7. Exclusion Reason: 1

Wright, A., Poon, E. G., Wald, J., Feblowitz, J., Pang, J. E., Schnipper, J. L., … Middleton, B. (2012). Randomized controlled trial of health maintenance reminders provided directly to patients through an electronic PHR. *Journal of General Internal Medicine*, *27*(1), 85–92. Exclusion Reason: 1

Ozanne, E. M., Howe, R., Omer, Z., & Esserman, L. J. (2014). Development of a personalized decision aid for breast cancer risk reduction and management. *BMC Medical Informatics And Decision Making*, *14*, 4. Exclusion Reason: 1

Vermeulen, J., Neyens, J. C. L., Spreeuwenberg, M. D., van Rossum, E., Sipers, W., Habets, H., … de Witte, L. P. (2013). User-centered development and testing of a monitoring system that provides feedback regarding physical functioning to elderly people. *Patient Preference and Adherence*, *7*, 843–854. Exclusion Reason: 2

Free, C., Whittaker, R., Knight, R., Abramsky, T., Rodgers, A., & Roberts, I. G. (2009). Txt2stop: a pilot randomised controlled trial of mobile phone-based smoking cessation support. *Tobacco Control*, *18*(2), 88–91. Exclusion Reason: 1

Wijsman, J., Grundlehner, B., Liu, H., Hermens, H., & Penders, J. (2011). Towards mental stress detection using wearable physiological sensors. In *Conference Proceedings: ... Annual International Conference Of The IEEE Engineering In Medicine And Biology Society. IEEE Engineering In Medicine And Biology Society. Annual Conference* (Vol. 2011, pp. 1798–1801). Faculty of Electrical Engineering, Mathematics and Computer Science, University of Twente Exclusion Reason: 3

Carrà, G., Crocamo, C., Bartoli, F., Carretta, D., Schivalocchi, A., Bebbington, P. E., & Clerici, M. (2016). Impact of a Mobile E-Health Intervention on Binge Drinking in Young People: The Digital-Alcohol Risk Alertness Notifying Network for Adolescents and Young Adults Project. *Journal of Adolescent Health*, *58*(5), 520–526. Exclusion Reason: 1

Colwell, N., Egan, S., Sheppard, A., & Walsh, K. (2015). Real time longitudinal monitoring of patient diet, exercise and weight loss has substantial positive effects on metabolic syndrome parameters. *Heart*, *101*, A23–A24. Exclusion Reason: 1

Fischer, G. S., Hess, R., Landeen, B. M., Weimer, M., Zieth, C. R., Dong, X., … Roberts, M. S. (2013). Electronic reminders to patients within an interactive patient health record. *Telemedicine Journal and E-Health : The Official Journal of the American Telemedicine Association*, *19*(6), 497–500. **Exclusion Reason: 1**

O'Hara, B. J., Phongsavan, P., Venugopal, K., Eakin, E. G., Eggins, D., Caterson, H., … Bauman, A. E. (2012). Effectiveness of Australia's Get Healthy Information and Coaching Service®: Translational research with population wide impact. *Preventive Medicine*, *55*(4), 292–298. **Exclusion Reason: 1**

Steinberg, D. M., Christy, J., Batch, B. C., Askew, S., Moore, R. H., Parker, P., & Bennett, G. G. (2017). Preventing Weight Gain Improves Sleep Quality Among Black Women: Results from a RCT. *Annals of Behavioral Medicine*, *51*(4), 555–566. **Exclusion Reason: 1**

Foucher-Urcuyo, J., Longworth, D., Roizen, M., & Rothberg, M. B. (2017). Patient-Entered Wellness Data and Tailored Electronic Recommendations Increase Preventive Care. *Journal of the American Board of Family Medicine*, *30*(3), 350–361. **Exclusion Reason: 1**

Li, X., Scruggs, P., Tan, J., He, H., & Yang, X. (2015). Design and development of smart sensor technology in a smart home for healthy independent living - A pilot study. In *IIE Annual Conference and Expo 2015* (pp. 3221–3226). Industrial and Systems Engineering, University of Tennessee, Knoxville, United States Mechanical, Aerospace and Biomedical Engineering, University of Tennessee, Knoxville, United States: Institute of Industrial Engineers. **Exclusion Reason: 1**

Gao, K., Boyd, C., Wiederhold, M. D., & Wiederhold, B. K. (2014). VR Mobile Solutions For Chronic Stress Reduction in Young Adults. *Studies In Health Technology And Informatics*, *199*, 88–93. **Exclusion Reason: 1**

Takeuchi, T., Fujii, T., Narumi, T., Tanikawa, T., & Hirose, M. (2015). Considering individual taste in social feedback to improve eating habits. In *iEEE*. **Exclusion Reason: 1**

Buist, D. S. M., Knight Ross, N., Reid, R. J., & Grossman, D. C. (2014). Electronic health risk assessment adoption in an integrated healthcare system. *The American Journal Of Managed Care*, *20*(1), 62–69. **Exclusion Reason: 1**

Conroy, M. B., Bhargava, T., Tudorascu, D., Karpov, I., Boan, J., Hess, R., … McTigue, K. M. (2012). Defining and predicting adherence to an online lifestyle program: 12-month results from the phit study. *Circulation*, *125*(10). **Exclusion Reason: 1**

Youm, S., & Park, S.-H. (2014). Development and evaluation of a mobile application for personal lifestyle check-up and improvement. *Telemedicine and E-Health*, *20*(11), 1057–1063. **Exclusion Reason: 1**

van den Brekel-Dijkstra, K., Rengers, A. H., Niessen, M. A. J., de Wit, N. J., & Kraaijenhagen, R. A. (2016). Personalized prevention approach with use of a web-based cardiovascular risk assessment with tailored lifestyle follow-up in primary care practice--a pilot study. *European Journal Of Preventive Cardiology*, *23*(5), 544–551. **Exclusion Reason: 1**

Balmford, J., Borland, R., & Benda, P. (2008). Patterns of use of an automated interactive personalized coaching program for smoking cessation. *Journal Of Medical Internet Research*, *10*(5), e54–e54. Exclusion Reason: 1

Boudreaux, E. D., Bedek, K. L., Byrne, N. J., Baumann, B. M., Lord, S. A., & Grissom, G. (2012). The Computer-Assisted Brief Intervention for Tobacco (CABIT) program: a pilot study. *Journal Of Medical Internet Research*, *14*(6), e163–e163. Exclusion Reason: 1

Hess, R., Fischer, G. S., Sullivan, S. M., Dong, X., Weimer, M., Zeith, C., … Roberts, M. S. (2014). Patterns of response to patient-centered decision support through a personal health record. *Telemedicine Journal and E-Health : The Official Journal of the American Telemedicine Association*, *20*(11), 984–989. Exclusion Reason: 1

Woolf, S. H., Krist, A. H., Johnson, R. E., Wilson, D. B., Rothemich, S. F., Norman, G. J., & Devers, K. J. (2006). "A Practice-Sponsored Web Site to Help Patients Pursue Healthy Behaviors: An ACORN Study": Corrections. *Annals of Family Medicine*, *4*(4), 371. Exclusion Reason: 1

Kaipainen, K., Mattila, E., Väätänen, A., Happonen, A. P., Korhonen, I., Kinnunen, M. L., … Lappalainen, R. (2010). Web, mobile and monitoring technologies in self-management of psychophysiological wellbeing: Usage and user experiences. In *IADIS International Conference e-Health 2010, EH, Part of the IADIS Multi Conference on Computer Science and Information Systems 2010, MCCSIS 2010* (pp. 167–177). VTT Technical Research Centre of Finland Exclusion Reason: 2

Ganju, N., Krapf, J., Benham, J., & Marko, K. I. (2016). Preventing excessive gestational weight gain by using connected weight scales paired with mobile apps. *Obstetrics and Gynecology*, *127*, 103S–104S. Exclusion Reason: 1

Schulz, D. N., Kremers, S. P. J., Vandelanotte, C., van Adrichem, M. J. G., Schneide, F., Candel, M. J. J. M., & de Vries, H. (2014). Effects of a Web-based tailored multiple-lifestyle intervention for adults: A two-year randomized controlled trial comparing sequential and simultaneous delivery modes. *Journal of Medical Internet Research*, *16*(1), 48–65. Exclusion Reason: 1

Direito, A., Jiang, Y., Whittaker, R., & Maddison, R. (2015). Apps for IMproving FITness and increasing physical activity among young people: The AIMFIT pragmatic randomized controlled trial. *Journal of Medical Internet Research*, *17*(8). Exclusion Reason: 4

Alpert, J. M., Krist, A. H., Aycock, R. A., & Kreps, G. L. (2017). Designing User-Centric Patient Portals: Clinician and Patients' Uses and Gratifications. *Telemedicine Journal and E-Health : The Official Journal of the American Telemedicine Association*, *23*(3), 248–253. Exclusion Reason: 1

Andrew, A., Borriello, G., & Fogarty, J. (2013). Simplifying Mobile Phone Food Diaries. *Proceedings of the ICTs for Improving Patients Rehabilitation Research Techniques*, 260–263. Exclusion Reason: 1
