# Supplementary material for: Electronic Patient-Generated Health Data to Facilitate Disease Prevention and Health Promotion: Scoping Review
Source: J Med Internet Res. 2019 Oct 14;21(10):e13320. doi: 10.2196/13320 (PMC6914107; doi:10.2196/13320)
Supplement: Multimedia Appendix 5 [file jmir_v21i10e13320_app5.pdf]

Multimedia Appendix: List and characteristics of included studies

Study number and study title

1. Aalbers, Teun, Li Qin, Maria A.E. Baars, Annet De Lange, Roy P.C. Kessels, and Marcel G.M.Olde Rikkert. “Changing Behavioral Lifestyle Risk Factors Related to Cognitive Decline in Later Life Using a Self-Motivated EHealth Intervention in Dutch Adults.” *Journal of Medical Internet Research* 18, no. 6 (2016): e171. doi:10.2196/jmir.5269.
2. Abroms, Lorien C, Meenakshi Ahuja, Yvonne Kodl, Lalida Thaweethai, Justin Sims, Jonathan Winickoff, and Richard A Windsor. “Text2Quit: Results from a Pilot Test of a Personalized, Interactive Mobile Health Smoking Cessation Program LORIEN.” *Journal Of Health Communication* 17, no. Suppl 1 (2012): 44–53. doi:10.1080/10810730.2011.649159.Text2Quit.
3. Abroms, Lorien C, Ashley L: Boal, Sauel J Simmens, Judith A Mendel, and Richard A Windsor. “A Randomized Trial of Text2Quit: A Text Messaging Program for Smoking Cessation.” *American Journal of Preventive Medicine* 47, no. 3 (2014): 242–50. doi:10.1016/j.amepre.2014.04.010.A.
4. Adams, Marc A, James F Sallis, Gregory J Norman, Melbourne F Hovell, Eric B Hekler, and Elyse Perata. “An Adaptive Physical Activity Intervention for Overweight Adults: A Randomized Controlled Trial.” *PLoS ONE* 8, no. 12 (2013): e82901. doi:10.1371/journal.pone.0082901.
5. Ahmed, A, and M Ouzzani. “Development and Assessment of an Interactive Web-Based Breastfeeding Monitoring System (LACTOR).” *Maternal and Child Health Journal* 17, no. 5 (2013): 809–15. doi:10.1007/s10995-012-1074-z.
6. Ahtinen, A., E. Mattila, A. Vaatanen, L. Hynninen, J. Salminen, E. Koskinen, and K. Laine. “User Experiences of Mobile Wellness Applications in Health Promotion.” In *Pervasive Computing Technologies for Healthcare, 2009. PervasiveHealth 2009. 3rd International Conference*, 1–8, 2009. doi:10.4108/ICST.PERVASIVEHEALTH2009.6007.
7. Ayubi, Soleh U Al, Bambang Parmanto, Robert Branch, and Dan Ding. “A Persuasive and Social MHealth Application for Physical Activity: A Usability and Feasibility Study.” *JMIR MHealth and UHealth* 2, no. 2 (2014): e25. doi:10.2196/mhealth.2902.
8. Al-Kuwari, Mohamed Ghaith, Abdulla Saeed Al-Mohannadi, Izzeldin Ibrahim El-Jack, and Fuad Almudahka. “Effect of Online Pedometer Program on Physical Activity in Qatar.” *The Journal Of Sports Medicine And Physical Fitness* 56, no. 3 (2016): 275–80.
9. Alessi, Sheila M, Carla J Rash, and Nancy M Petry. “A Randomized Trial of Adjunct MHealth Abstinence Reinforcement With Transdermal Nicotine and Counseling for Smoking Cessation.” *Nicotine & Tobacco Research* 19, no. 3 (2016): 290–98. doi:10.1093/ntr/ntw155.
10. Alessi, Sheila M., and Nancy M. Petry. “A Randomized Study of Cell Phone Technology to Reinforce Alcohol Abstinence in the Natural Environment.” *Addiction* 108, no. 5 (2013): 900–909. doi:10.1111/add.12093.A.
11. An, L C, M R S Demers, M A Kirch, S Considine-Dunn, V Nair, K Dasgupta, N Narisetty, K Resnicow, and J Ahluwalia. “A Randomized Trial of an Avatar-Hosted Multiple Behavior Change Intervention for Young Adult Smokers.” *Journal of National Cancer Institute* 47 (2013): 209–15. doi:10.1093/jncimonographs/lgt021.
12. Anton, Stephen D, Eric LeBlanc, H Raymond Allen, Christy Karabetian, Frank Sacks, George Bray, and Donald A Williamson. “Use of a Computerized Tracking System to

Monitor and Provide Feedback on Dietary Goals for Calorie-Restricted Diets: The POUNDS LOST Study.” *Journal of Diabetes Science and Technology* 6, no. 5 (September 2012): 1216–25. doi:10.1177/193229681200600527.

13. Attwood, S, H Parke, J Larsen, and K L Morton. “Using a Mobile Health Application to Reduce Alcohol Consumption: A Mixed-Methods Evaluation of the Drinkaware Track & Calculate Units Application.” *BMC Public Health* 17, no. 1 (2017): 394. doi:10.1186/s12889-017-4358-9.
14. Beasley, Jeannette M, William T Riley, Amanda Davis, and Jatinder Singh. “Evaluation of a PDA-Based Dietary Assessment and Intervention Program : A Randomized Controlled Trial.” *Journal of the American College of Nutrition* 27, no. 2 (2008): 280–86.
15. Bertz, Fredrik, Carly R Pacanowski, and David A Levitsky. “Frequent Self-Weighing with Electronic Graphic Feedback to Prevent Age-Related Weight Gain in Young Adults.” *Obesity* 23, no. 10 (2015): 2009–14. doi:10.1002/oby.21211.Frequent.
16. Blomfield, Rebecca L, Clare E Collins, Melinda J Hutchesson, Myles D Young, Megan E Jensen, Robin Callister, and Philip J Morgan. “Impact of Self-Help Weight Loss Resources with or without Online Support on the Dietary Intake of Overweight and Obese Men: The SHED-IT Randomised Controlled Trial.” *Obesity Research and Clinical Practice* 8, no. 5 (2014): e476–87. doi:10.1016/j.orcp.2013.09.004.
17. Bond, Dale S, J Graham Thomas, Hollie A Raynor, Jon Moon, Jared Sieling, Jennifer Trautvetter, Tiffany Leblond, and Rena R Wing. “B-MOBILE - A Smartphone-Based Intervention to Reduce Sedentary Time in Overweight/Obese Individuals: A within-Subjects Experimental Trial.” *PLoS ONE* 9, no. 6 (2014): e100821. doi:10.1371/journal.pone.0100821.
18. Bove, Alfred A., William P. Santamore, Carol Homko, Abul Kashem, Robert Cross, Timothy R. McConnell, Gail Shirk, and Francis Menapace. “Reducing Cardiovascular Disease Risk in Medically Underserved Urban and Rural Communities.” *American Heart Journal* 161, no. 2 (2011): 351–59. doi:10.1016/j.ahj.2010.11.008.
19. Brendryen, H, I O Lund, A B Johansen, M Riksheim, S Nesvåg, and F Duckert. “Balance--a Pragmatic Randomized Controlled Trial of an Online Intensive Self-Help Alcohol Intervention.” *Addiction* 109, no. 2 (2014): 218–26. doi:10.1111/add.12383.
20. Brindal, Emily, Jill Freyne, Ian Saunders, Shlomo Berkovsky, Greg Smith, and Manny Noakes. “Features Predicting Weight Loss in Overweight or Obese Participants in a Web-Based Intervention : Randomized Trial Corresponding Author :” *Journal of MEdical Inter* 14, no. 6 (2012): e173. doi:10.2196/jmir.2156.
21. Burke, Lora E, Mindi a Styn, Susan M Sereika, Molly B Conroy, Lei Ye, Karen Glanz, Mary Ann Sevick, and Linda J Ewing. “Using MHealth Technology to Enhance Self-Monitoring for Weight Loss A Randomized Trial.” *American Journal of Preventive Medicine* 43, no. 1 (2013): 20–26. doi:10.1016/j.amepre.2012.03.016.Using.
22. Burke, Lora E, Yaguang Zheng, Qianheng Ma, Juliet Mancino, Edvin Music, Mindi Styn, Linda Ewing, et al. “The SMARTER Pilot Study : Testing Feasibility of Real-Time Feedback for Dietary Self-Monitoring.” *Preventive Medicine Reports* 6 (2017): 278–85. doi:10.1016/j.pmedr.2017.03.017.
23. Butryn, M L, D Arigo, G A Raggio, M Colasanti, and E M Forman. “Enhancing Physical Activity Promotion in Midlife Women with Technology-Based Self-Monitoring and Social Connectivity: A Pilot Study.” *Journal of Health Psychology* 21, no. 8 (2016): 1548–55. doi:10.1177/1359105314558895.
24. Cadmus-bertram, Lisa A, Bess H Marcus, Ruth E Patterson, Barbara A Parker, and Brittany L Morey. “Randomized Trial of a Fitbit-Based Physical Activity Intervention

for Women." *American Journal of Preventive Medicine* 49, no. 3 (2015): 414–18. doi:10.1016/j.amepre.2015.01.020.Randomized.
25. Cadmus-Bertram, Lisa, Julie B Wang, Ruth E Patterson, Vicky A Newman, Barbara A Parker, and John P Pierce. "Web-based Self-monitoring for Weight Loss among Overweight/Obese Women at Increased Risk for Breast Cancer: The HELP Pilot Study." *Psycho-Oncology* 22, no. 8 (2013): 1821–28. jppierce@ucsd.edu.
26. Carels, Robert A, Jennifer C Selensky, James Rossi, Chelsey Solar, and Reid Hlavka. "A Novel Stepped-Care Approach to Weight Loss: The Role of Self-Monitoring and Health Literacy in Treatment Outcomes." *Eating Behaviors* 26 (August 2017): 76–82. doi:10.1016/j.eatbeh.2017.01.009.
27. Carr, Lucas J, Beth Lewis, Sheri Hartman, Gregory Dominick, and Bess H Marcus. "Randomized Controlled Trial Testing an Internet Physical Activity Intervention for Sedentary Adults." *Health Psychology* 32, no. 3 (2013): 328–36. doi:10.1037/a0028962.Randomized.
28. Carter, Michelle Clare, Victoria Jane Burley, Camilla Nykjaer, and Janet Elizabeth Cade. "Adherence to a Smartphone Application for Weight Loss Compared to Website and Paper Diary: Pilot Randomized Controlled Trial." *Journal of Medical Internet Research* 15, no. 4 (2013): e32. doi:10.2196/jmir.2283.
29. Cavallo, David N, Deborah F Tate, Amy V Ries, Jane D Brown, Robert F Devellis, and Alice S Ammerman. "A Social Media–Based Physical Activity Intervention: A Randomized Controlled Trial." *American Journal of Preventive Medicine* 43, no. 5 (2012): 527–32. doi:10.1016/j.amepre.2012.07.019.A.
30. Chambliss, Heather O, Rachel C Huber, Carrie E Finley, Scott O McDoniel, Heather Kitzman-Ulrich, and William J Wilkinson. "Computerized Self-Monitoring and Technology-Assisted Feedback for Weight Loss with and without an Enhanced Behavioral Component." *Patient Education and Counseling* 85, no. 3 (2011): 375–82. hchmblss@memphis.edu.
31. Chan, Catherine B, Daniel A J Ryan, and Catrine Tudor-Locke. "Health Benefits of a Pedometer-Based Physical Activity Intervention in Sedentary Workers." *Preventive Medicine* 39, no. 6 (2004): 1215–22. doi:10.1016/j.ypmed.2004.04.053.
32. Choe, Eun Kyoung. "Designing Self-Monitoring Technology to Promote Data Capture and Reflection." *Dissertation Abstracts International Section A: Humanities and Social Sciences*. University of Washington, 2014. http://gateway.proquest.com/openurl?url_ver=Z39.88-2004&rft_val_fmt=info:ofi/fmt:kev:mtx:dissertation&res_dat=xri:pqm&rft_dat=xri:pqdiss:3641503%5Cnhttp://ovidsp.ovid.com/ovidweb.cgi?T=JS&PAGE=reference&D=psyc11&NEWS=N&AN=2015-99150-008.
33. Choi, Ji Won, Ji hyeon Lee, Eric Vittinghoff, and Yoshimi Fukuoka. "MHealth Physical Activity Intervention: A Randomized Pilot Study in Physically Inactive Pregnant Women." *Maternal and Child Health Journal* 20, no. 5 (2016): 1091–1101. doi:10.1007/s10995-015-1895-7.
34. Chou, Ann F, Zsolt Nagykaldi, Cheryl B Aspy, and James W Mold. "Promoting Patient-Centered Preventive Care Using a Wellness Portal: Preliminary Findings." *Journal Of Primary Care & Community Health* 1, no. 2 (2010): 88–92. doi:10.1177/2150131910365358.
35. Chung, A E, A C Skinner, S E Hasty, and E M Perrin. "Tweeting to Health: A Novel MHealth Intervention Using Fitbits and Twitter to Foster Healthy Lifestyles." *Clinical Pediatrics* 56, no. 1 (2017): 26–32. doi:10.1177/0009922816653385.
36. Chung, Louisa Ming Yan, Queenie Pui Sze Law, Shirley Siu Ming Fong, and Joanne Wai Yee Chung. "Teledietetics Improves Weight Reduction by Modifying Eating

Behavior: A Randomized Controlled Trial." *Telemedicine and E-Health* 20, no. 1 (2014): 55–62. doi:10.1089/tmj.2013.0104.

37. Collins, Clare E, Philip J Morgan, Penelope Jones, Kate Fletcher, Julia Martin, Elroy J Aguiar, Ashlee Lucas, Melinda J Neve, and Robin Callister. "A 12-Week Commercial Web-Based Weight-Loss Program for Overweight and Obese Adults: Randomized Controlled Trial Comparing Basic Versus Enhanced Features." *Journal of Medical Internet Research* 14, no. 2 (2012): e57. doi:10.2196/jmir.1980.
38. Collinson, A, R Lindley, A Campbell, I Waters, T Lindley, and A Wallace. "An Evaluation of an Internet-Based Approach to Weight Loss with Low Glycaemic Load Principles." *Journal of Human Nutrition and Dietetics* 24 (2011): 192–95. doi:10.1111/j.1365-277X.2010.01138.x.
39. Compernolle, Sofie, Corneel Vandelanotte, Greet Cardon, Ilse De Bourdeaudhuij, and Katrien De Cocker. "Effectiveness of a Web-Based, Computer-Tailored, Pedometer-Based Physical Activity Intervention for Adults: A Cluster Randomized Controlled Trial." *Journal of Medical Internet Research* 17, no. 2 (2015): e38. doi:10.2196/jmir.3402.
40. Comulada, W Scott, Dallas Swendeman, Maryann K Koussa, Deborah Mindry, Melissa Medich, Deborah Estrin, Neil Mercer, and Nithya Ramanathan. "Adherence to Self-Monitoring Healthy Lifestyle Behaviours through Mobile Phone-Based Ecological Momentary Assessments and Photographic Food Records over 6 Months in Mostly Ethnic Minority Mothers." *Public Health Nutrition* 21, no. 4 (2017): 679–88. doi:10.1017/S1368980017003044.
41. Consolvo, Sunny, Katherine Everitt, Ian Smith, and James A Landay. "Design Requirements for Technologies That Encourage Physical Activity." In *Proceedings of the SIGCHI Conference on Human Factors in Computing Systems - CHI '06*, 457–66, 2006. doi:10.1145/1124772.1124840.
42. Cordeiro, Felicia, Elizabeth Bales, Erin Cherry, and James Fogarty. "Rethinking the Mobile Food Journal: Exploring Opportunities for Leightweight Photo-Based Capture." In *Proceedings of the 33rd Annual ACM Conference on Human Factors in Computing Systems - CHI '15*, 3207–16, 2015. doi:10.1145/2702123.2702154.
43. Crane, D, C Garnett, J Brown, R West, and S Michie. "Factors Influencing Usability of a Smartphone App to Reduce Excessive Alcohol Consumption: Think Aloud and Interview Studies." *Frontiers in Public Health* 5 (2017): 1–19. doi:10.3389/fpubh.2017.00039.
44. Dallery, Jesse, Irene M Glenn, and Bethany R Raiff. "An Internet-Based Abstinence Reinforcement Treatment for Cigarette Smoking." *Drug and Alcohol Dependence* 86, no. 2–3 (2006): 1–9. doi:10.1016/j.drugalcdep.2006.06.013.
45. Dallery, Jesse, Steven Meredith, Brantley Jarvis, and Paul A Nuzzo. "Internet-Based Group Contingency Management to Promote Smoking Abstinence." *Experimental and Clinical Psychopharmacology* 23, no. 3 (2015): 176–83. doi:10.1186/s40945-017-0033-9.Using.
46. Darlow, S, and C Heckman. "Results From a Tailored SMS and Behavior-Tracking Pilot Study on Sun-Safe Behaviors in Young Women." *Health Education & Behavior* 44, no. 6 (2017): 937–44. doi:10.1177/1090198117699507.
47. Davy, Brenda M, Kerry L Potter, Elizabeth A Dennis Parker, Samantha Harden, Jennie L Hill, Tanya M Halliday, and Paul A Estabrooks. "Feasibility , Effectiveness , and Perceptions of an Internet- and Incentive-Based Behavioral Weight Loss Intervention for Overweight and Obese College Freshmen : A Mixed Methods Approach." *Open Journal of Preventive Medicine* 3, no. 7 (2013): 429–40.

48. Duncan, Mitch, Corneel Vandelanotte, Gregory S. Kolt, Richard R. Rosenkranz, Cristina M. Caperchione, Emma S. George, Hang Ding, et al. "Effectiveness of a Web- and Mobile Phone-Based Intervention to Promote Physical Activity and Healthy Eating in Middle-Aged Males: Randomized Controlled Trial of the ManUp Study." *Journal of Medical Internet Research* 16, no. 6 (2014): e136. doi:10.2196/jmir.3107.
49. Eccher, Claudio, Maria Botteri, Daniele Ortolani, Stefano Forti, and Enrico Maria Piras. "A Mobile Logbook to Diagnose Masked Hypertension: A Pilot Application." In *MIE*, 363–67, 2014. doi:10.3233/978-1-61499-432-9-363.
50. Eisenhauer, Christine M., Patricia A. Hageman, Sheri Rowland, Betsy J. Becker, Susan A. Barnason, and Carol H. Pullen. "Acceptability of MHealth Technology for Self-Monitoring Eating and Activity among Rural Men." *Public Health Nursing* 34, no. 2 (2017): 138–46. doi:10.1111/phn.12297.
51. Finkelstein, Eric A., Benjamin A. Haaland, Marcel Bilger, Aarti Sahasranaman, Robert A. Sloan, Ei Ei Khaing Nang, and Kelly R. Evenson. "Effectiveness of Activity Trackers with and without Incentives to Increase Physical Activity (TRIPPA): A Randomised Controlled Trial." *The Lancet Diabetes and Endocrinology* 4, no. 12 (2016): 983–95. doi:10.1016/S2213-8587(16)30284-4.
52. Foster, Derek, Conor Linehan, Ben Kirman, Shaun Lawson, and Gary James. "Motivating Physical Activity at Work : Using Persuasive Social Media for Competitive Step Counting." In *Proceedings of the 14th International Academic MindTrek Conference: Envisioning Future Media Environments*, 111–16, 2010.
53. Freak-poli, Rosanne, Rory Wolfe, Kathryn Backholer, Maximilian De Courten, and Anna Peeters. "Impact of a Pedometer-Based Workplace Health Program on Cardiovascular and Diabetes Risk pro Fi Le." *Preventive Medicine* 53, no. 3 (2011): 162–71. doi:10.1016/j.ypmed.2011.06.005.
54. Frisch, Sabine, Armin Zittermann, Heiner K. Berthold, Christian Götting, Joachim Kuhn, Knut Kleesiek, Peter Stehle, and Heinrich Körtke. "A Randomized Controlled Trial on the Efficacy of Carbohydrate-Reduced or Fat-Reduced Diets in Patients Attending a Telemedically Guided Weight Loss Program." *Cardiovascular Diabetology* 8 (2009): 1–10. doi:10.1186/1475-2840-8-36.
55. Fukuoka, Yoshimi, Caryl L Gay, Kevin L Joiner, and Eric Vittinghoff. "A Novel Diabetes Prevention Intervention Using a Mobile App: A Randomized Controlled Trial With Overweight Adults at Risk." *American Journal of Preventive Medicine* 49, no. 2 (2015): 223–37. doi:10.1002/stem.1868.Human.
56. Fukuoka, Yoshimi, Teri Lindgren, and Soson Jong. "Qualitative Exploration of the Acceptability of a Mobile Phone and Pedometer-Based Physical Activity Program in a Diverse Sample of Sedentary Women." *Public Health Nursing* 29, no. 3 (2012): 232–40. doi:10.1111/j.1525-1446.2011.00997.x.
57. Fukuoka, Yoshimi, Eric Vittinghoff, So Son Jong, and William Haskell. "Innovation to Motivation - Pilot Study of a Mobile Phone Intervention to Increase Physical Activity among Sedentary Women Yoshimi." *Preventive Medicine* 51, no. 3–4 (2011): 287–89. doi:10.1016/j.ypmed.2010.06.006.Innovation.
58. Gaggioli, Andrea, Pietro Cipresso, Silvia Serino, Danilo Marco Campanaro, Federica Pallavicini, Brenda K Wiederhold, and Giuseppe Riva. "Positive Technology: A Free Mobile Platform for the Self-Management of Psychological Stress." *Annual Review of CyberTherapy and Telemedicine* 12 (2014): 25–29. http://search.ebscohost.com/login.aspx?direct=true&db=cmedm&AN=24875684&site=ehost-live.

59. Gajecki, Mikael, Anne H Berman, Kristina Sinadinovic, Ingvar Rosendahl, and Claes Andersson. “Mobile Phone Brief Intervention Applications for Risky Alcohol Use among University Students : A Randomized Controlled Study.” *Addiction Science & Clinical Practice* 9, no. 11 (2014): 1–12. doi:10.1186/1940-0640-9-11.
60. Ganesan, Anand N., Jennie Louise, Matthew Horsfall, Shane A. Bilsborough, Jeroen Hendriks, Andrew D. McGavigan, Joseph B. Selvanayagam, and Derek P. Chew. “International Mobile-Health Intervention on Physical Activity, Sitting, and Weight: The Stepathlon Cardiovascular Health Study.” *Journal of the American College of Cardiology* 67, no. 21 (2016): 2453–63. doi:10.1016/j.jacc.2016.03.472.
61. Gasser, Roland, Dominique Brodbeck, Markus Degen, Jürg Luthiger, Remo Wyss, and Serge Reichlin. “Persuasiveness of a Mobile Lifestyle Coaching Application Using Social Facilitation.” In *International Conference on Persuasive Technology*, 27–28, 2006.
62. Glanz, K, S Murphy, J Moylan, D Evensen, and J D Curb. “Improving Dietary Self-Monitoring and Adherence With Hand-Held Computers: A Pilot Study.” *American Journal of Health Promotion* 20, no. 3 (2006): 165–70. doi:10.4278/0890-1171-20.3.165.
63. Glynn, Liam G, Patrick S Hayes, Monica Casey, Fergus Glynn, Alberto Alvarez-Iglesias, John Newell, Gearóid Olaighin, David Heaney, Martin O’Donnell, and Andrew W Murphy. “Effectiveness of a Smartphone Application to Promote Physical Activity in Primary Care: The SMART MOVE Randomised Controlled Trial.” *The British Journal Of General Practice* 64, no. 624 (2014): e384–91. doi:10.3399/bjgp14X680461.
64. González, Cintia, Pau Herrero, José M. Cubero, José M. Iniesta, M. Elena Hernando, Gema García-Sáez, Alvaro J. Serrano, et al. “PREDIRCAM EHealth Platform for Individualized Telemedical Assistance for Lifestyle Modification in the Treatment of Obesity, Diabetes, and Cardiometabolic Risk Prevention: A Pilot Study (PREDIRCAM 1).” *Journal of Diabetes Science and Technology* 7, no. 4 (2013): 888–97. doi:10.1177/193229681300700411.
65. Gotsis, Marientina, Hua Wang, Donna Spruijt-Metz, Maryalice Jordan-Marsh, and Thomas William Valente. “Wellness Partners : Design and Evaluation of a Web-Based Physical Activity Diary with Social Gaming Features for Adults.” *JMIR Rese* 2, no. 1 (n.d.): e10. doi:10.2196/resprot.2132.
66. Goulis, D G, G D Giaglis, S A Boren, I Lekka, E Bontis, E A Balas, N Maglaveras, and A Avramides. “Effectiveness of Home-Centered Care through Telemedicine Applications for Overweight and Obese Patients: A Randomized Controlled Trial.” *International Journal of Obesity* 28, no. 11 (2004): 1391–98. doi:10.1038/sj.ijo.0802773.
67. Gouveia, Rúben, Evangelos Karapanos, and Marc Hassenzahl. “How Do We Engage With Activity Trackers? A Longitudinal Study of Habito.” In *UbiComp*, 2–5, 2015. doi:10.1145/2750858.2804290.
68. Gow, Rachel W, Sara E Trace, and Suzanne E Mazzeo. “Preventing Weight Gain in First Year College Students: An Online Intervention to Prevent the ‘Freshman Fifteen.’” *Eating Behaviors* 11, no. 1 (2010): 33–39. doi:10.1016/j.eatbeh.2009.08.005.Preventing.
69. Greene, Jessica, Rebecca Sacks, Brigitte Piniewski, David Kil, and Jin S. Hahn. “The Impact of an Online Social Network With Wireless Monitoring Devices on Physical Activity and Weight Loss.” *Journal of Primary Care and Community Health* 4, no. 3 (2013): 189–94. doi:10.1177/2150131912469546.

70. Haapala, Irja, C Barengo, Simon Biggs, Leena Surakka, and Pirjo Manninen. “Weight Loss by Mobile Phone : A 1-Year Effectiveness Study.” *Public Health Nutrition* 12, no. 12 (2009): 2382–91. doi:10.1017/S1368980009005230.
71. Hargreaves, Elaine Anne, Nanette Mutrie, and Jade Dallas Fleming. “A Web-Based Intervention to Encourage Walking (StepWise): Pilot Randomized Controlled Trial.” *JMIR Research Protocols* 5, no. 1 (2016): e14. doi:10.2196/resprot.4288.
72. Hartman, Sheri J, Sandahl H Nelson, Lisa A Cadmus-bertram, E Ruth, Barbara A Parker, and John P Pierce. “Technology- and Phone-Based Weight Loss Intervention: Pilot RCT in Women at Elevated Breast Cancer Risk.” *American Journal of Preventive Medicine* 51, no. 5 (2016): 714–21. doi:10.1016/j.amepre.2016.06.024.Technology-.
73. Harvey-berino, Jean, Stephen Pintauro, Paul Buzzell, and Elizabeth Casey Gold. “Effect of Internet Support on the Long-Term Maintenance of Weight Loss.” *Obesity Research* 12, no. 2 (2004): 320–29.
74. Harvey-Berino, Jean, Lizzy Pope, Beth Casey Gold, Heather Leonard, and Cynthia Belliveau. “Undergrad and Overweight : An Online Behavioral Weight Management Program for College Students.” *Journal of Nutrition Education and Behavior* 44, no. 6 (2012): 604–8. doi:10.1016/j.jneb.2012.04.016.
75. Hebden, L., A. Cook, H. P. van der Ploeg, L. King, A. Bauman, and M. Allman-Farinelli. “A Mobile Health Intervention for Weight Management among Young Adults: A Pilot Randomised Controlled Trial.” *Journal of Human Nutrition and Dietetics* 27, no. 4 (2014): 322–32. doi:10.1111/jhn.12155.
76. Henkemans, Olivier A Blanson, Paul J M Van Der Boog, Jasper Lindenberg, Charles A P G van der Mast, Mark A Neerincx, and Bertie J H M Sweetsloot-Schonk. “An Online Lifestyle Diary with a Persuasive Computer Assistant Providing Feedback On.” *Technology and Health Care* 17 (2009): 1–15. doi:10.3233/THC-2009-0545.
77. Herman, Christopher W., Shirley Musich, Chifung Lu, Stewart Sill, Joyce M. Young, and Dee W. Edington. “Effectiveness of an Incentive-Based Online Physical Activity Intervention on Employee Health Status.” *Journal of Occupational and Environmental Medicine* 48, no. 9 (2006): 889–95. doi:10.1097/01.jom.0000232526.27103.71.
78. Hezarjaribi, Niloofar, Sepideh Mazrouee, and Hassan Ghasemzadeh. “Speech2Health: A Mobile Framework for Monitoring Dietary Composition From Spoken Data.” *IEEE Journal Of Biomedical And Health Informatics* 22, no. 1 (2018): 252–64. doi:10.1109/JBHI.2017.2709333.
79. Hunter, Christine M, Alan L Peterson, Lisa M Alvarez, Walker C Poston, Antoinette R Brundige, C Keith Haddock, David L Van Brunt, and John P Foreyt. “Weight Management Using the Internet A Randomized Controlled Trial.” *American Journal of Preventive Medicine* 34, no. 2 (2008): 119–26. doi:10.1016/j.amepre.2007.09.026.
80. Hurling, Robert, Michael Catt, Marco De Boni, Bruce William Fairley, Tina Hurst, Peter Murray, Alannah Richardson, and Jaspreet Singh Sodhi. “Using Internet and Mobile Phone Technology to Deliver an Automated Physical Activity Program: Randomized Controlled Trial.” *Journal of Medical Internet Research* 9, no. 2 (2007): e7. doi:10.2196/jmir.9.2.e7.
81. Hutchesson, Melinda J, Megan E Rollo, Robin Callister, and Clare E Collins. “Self-Monitoring of Dietary Intake by Young Women: Online Food Records Completed on Computer or Smartphone Are as Accurate as Paper-Based Food Records but More Acceptable.” *Journal of the Academy of Nutrition and Dietetics* 115, no. 1 (2015): 87–94. doi:10.1016/j.jand.2014.07.036.

82. Ipjian, Michelle L, and Carol S Johnston. “Smartphone Technology Facilitates Dietary Change in Healthy Adults.” *Nutrition* 33 (2017): 343–47. doi:10.1016/j.nut.2016.08.003.
83. Jacobs, Stephanie. “Increasing Self-Monitoring Adherence Using Smartphone Technology to Facilitate Weight Loss.” Fairleigh Dickinson University, 2014.
84. Jauho, Anna Maiju, Riitta Pyky, Riikka Ahola, Maarit Kangas, Paula Virtanen, Raija Korpelainen, and Timo Jämsä. “Effect of Wrist-Worn Activity Monitor Feedback on Physical Activity Behavior: A Randomized Controlled Trial in Finnish Young Men.” *Preventive Medicine Reports* 2 (2015): 628–34. doi:10.1016/j.pmedr.2015.07.005.
85. Johnson, Fiona, and Jane Wardle. “The Association between Weight Loss and Engagement with a Web-Based Food and Exercise Diary in a Commercial Weight Loss Programme: A Retrospective Analysis.” *International Journal of Behavioral Nutrition and Physical Activity* 8, no. 83 (2011): 1–7. doi:10.1186/1479-5868-8-83.
86. Jones, Erin M. “Electronic Apps for Food and Appetite Monitoring: Acceptability and Reactive Effects in Women with Eating and Weight Concerns.” Emory University, 2007.
87. Karppinen, Pasi, Harri Oinas-Kukkonen, Tuomas Alahäivälä, Terhi Jokelainen, Anna-Maria Keränen, Tuire Salonurmi, and Markku Savolainen. “Persuasive User Experiences of a Health Behavior Change Support System: A 12-Month Study for Prevention of Metabolic Syndrome.” *International Journal of Medical Informatics* 96 (2016): 51–61. doi:10.1016/j.ijmedinf.2016.02.005.
88. Kato-Lin, Yi-Chin, Rema Padman, Julie Downs, and Vibhanshu Abhishek. “Evaluating Consumer M-Health Services for Promoting Healthy Eating: A Randomized Field Experiment.” In *AMIA ... Annual Symposium Proceedings. AMIA Symposium*, 1947–56. Carnegie Mellon University, Pittsburgh, PA., 2015. http://search.ebscohost.com/login.aspx?direct=true&db=cmedm&AN=26958294&site=ehost-live.
89. Kendzor, Darla E., Kerem Shuval, Kelley Pettee Gabriel, Michael S. Businelle, Ping Ma, Robin R. High, Erica L. Cuate, et al. “Impact of a Mobile Phone Intervention to Reduce Sedentary Behavior in a Community Sample of Adults: A Quasi-Experimental Evaluation.” *Journal Of Medical Internet Research* 18, no. 1 (2016): e19. doi:10.2196/jmir.5137.
90. Kerr, Deborah A, Amelia J Harray, Christina M Pollard, Satvinder S Dhaliwal, Edward J Delp, Peter A Howat, Mark R Pickering, et al. “The Connecting Health and Technology Study: A 6-Month Randomized Controlled Trial to Improve Nutrition Behaviours Using a Mobile Food Record and Text Messaging Support in Young Adults.” *International Journal of Behavioral Nutrition and Physical Activity* 13, no. 52 (2016): 1–14. doi:10.1186/s12966-016-0376-8.
91. Kim, Heewon, Meara Faw, and Andreas Michaelides. “Mobile But Connected: Harnessing the Power of Self-Efficacy and Group Support for Weight Loss Success through MHealth Intervention.” *Journal of Health Communication* 22, no. 5 (2017): 395–402. doi:10.1080/10810730.2017.1296510.
92. Kim, Jeongeun. “A Qualitative Analysis of User Experiences With a Self-Tracker for Activity, Sleep, and Diet.” *Journal of Medical Internet Research* 3, no. 1 (2014): e8. doi:10.2196/ijmr.2878.
93. Kim, Ju Young, Nathan E Wineinger, Michael Taitel, Jennifer M Radin, Osayi Akinbosoye, Jenny Jiang, Nima Nikzad, Gregory Orr, Eric Topol, and Steve Steinhubl. “Self-Monitoring Utilization Patterns Among Individuals in an Incentivized Program for Healthy Behaviors.” *Journal Of Medical Internet Research* 18, no. 11 (2016): e292.

http://search.ebscohost.com/login.aspx?direct=true&db=cmedm&AN=27856407&site=ehost-live.
94. King, A C, E B Hekler, L A Grieco, S J Winter, J L Sheats, M P Buman, B Banerjee, T N Robinson, and J Cirimele. “Harnessing Different Motivational Frames via Mobile Phones to Promote Daily Physical Activity and Reduce Sedentary Behavior in Aging Adults.” *PLoS ONE* 8, no. 4 (2013): e62613. doi:10.1371/journal.pone.0062613.
95. Kirwan, Morwenna, Mitch J Duncan, Corneel Vandelanotte, and W Kerry Mummery. “Using Smartphone Technology to Monitor Physical Activity in the 10,000 Steps Program: A Matched Case–Control Trial.” *Journal Of Medical Internet Research* 14, no. 2 (April 2012): e55. doi:10.2196/jmir.1950.
96. Knight-Agarwal, Catherine, Deborah Lee Davis, Lauren Williams, Rachel Davey, Robert Cox, and Adam Clarke. “Development and Pilot Testing of the Eating4two Mobile Phone App to Monitor Gestational Weight Gain.” *JMIR MHealth and UHealth* 3, no. 2 (2015): e44. doi:10.2196/mhealth.4071.
97. Knight, Emily, Melanie I. Stuckey, and Robert J. Petrella. “Health Promotion Through Primary Care: Enhancing Self-Management With Activity Prescription and MHealth.” *Physician and Sportsmedicine* 42, no. 3 (2014): 90–99. doi:10.3810/psm.2014.09.2080.
98. Kraushaar, L E, and A Krämer. “Web-Enabled Feedback Control Over Energy Balance Promotes an Increase in Physical Activity and a Reduction of Body Weight and Disease Risk in Overweight Sedentary Adults.” *Prevention Science* 15, no. 4 (2014): 579–87. doi:10.1007/s11121-013-0398-2.
99. Krukowski, Rebecca A, Jean Harvey-Berino, Zoran Bursac, Taka Ashikaga, and Delia West. “Patterns of Success: Online Self-Monitoring in a Web-Based Behavioral Weight Control Program.” *Health Psychology* 32, no. 2 (2013): 164–70. doi:10.1586/14737175.2015.1028369.Focused.
100. Laing, Brian Y, Carol M Mangione, Chi-Hong Tseng, Mei Leng, Ekaterina Vaisberg, Megha Mahida, Michelle Bholat, Eve Glazier, Donald E Morisky, and Douglas S Bell. “Effectiveness of a Smartphone Application for Weight Loss Compared to Usual Care in Overweight Primary Care Patients: A Randomized Controlled Trial.” *Annals of Internal Medicine* 161, no. 10 0 (2015): S5-12. doi:10.7326/M13-3005.Effectiveness.
101. Lane, Nicholas D, Mu Lin, Mashfiqui Mohammod, Xiaochao Yang, Hong Lu, Giuseppe Cardone, Shahid Ali, et al. “BeWell : Sensing Sleep, Physical Activities and Social Interactions to Promote Wellbeing.” *Mobile N* 19 (2014): 345–59. doi:10.1007/s11036-013-0484-5.
102. Lara, J, N O’Brien, A Godfrey, B Heaven, E H Evans, S Lloyd, S Moffatt, et al. “Pilot Randomised Controlled Trial of a Web-Based Intervention to Promote Healthy Eating, Physical Activity and Meaningful Social Connections Compared with Usual Care Control in People of Retirement Age Recruited from Workplaces.” *PloS One* 11, no. 7 (2016): e0159703. doi:10.1371/journal.pone.0159703.
103. Leinonen, Anna-Maiju, Riitta Pyky, Riikka Ahola, Maarit Kangas, Pekka Siirtola, Tim Luoto, Heidi Enwald, et al. “Feasibility of Gamified Mobile Service Aimed at Physical Activation in Young Men: Population-Based Randomized Controlled Study (MOPO).” *Jmir Mhealth and Uhealth* 5, no. 10 (October 2017): e146. doi:10.2196/mhealth.6675.
104. Lin, Pao-Hwa, Stephen Intille, Gary Bennett, Hayden B Bosworth, Leonor Corsino, Corrine Voils, Steven Grambow, et al. “Adaptive Intervention Design in Mobile Health: Intervention Design and Development in the Cell Phone Intervention

for You (CITY) Trial Pao-Hwa.” *Clinical Tria* 12, no. 6 (2015): 634–45. doi:10.1002/stem.1868.Human.
105. Lyden, Jennifer R, Susan L Zickmund, Tina D Bhargava, Cindy L Bryce, Molly B Conroy, Gary S Fischer, Rachel Hess, Laurey R Simkin-Silverman, and Kathleen M McTigue. “Implementing Health Information Technology in a Patient-Centered Manner: Patient Experiences With an Online Evidence-Based Lifestyle Intervention.” *Journal For Healthcare Quality* 35, no. 5 (2013): 47–57. doi:10.1111/jhq.12026.
106. Maher, Carol, Monika Ferguson, Corneel Vandelanotte, Ron Plotnikoff, Ilse De Bourdeaudhuij, Samantha Thomas, Karen Nelson-Field, and Tim Olds. “A Web-Based, Social Networking Physical Activity Intervention for Insufficiently Active Adults Delivered via Facebook App: Randomized Controlled Trial.” *Journal of Medical Internet Research* 17, no. 7 (2015): e174. doi:10.2196/jmir.4086.
107. Mann, Davina, Lynn Riddell, Karen Lim, Linda K Byrne, Caryl Nowson, Manuela Rigo, Ewa A Szymlek-Gay, and Alison O Booth. “Mobile Phone App Aimed at Improving Iron Intake and Bioavailability in Premenopausal Women: A Qualitative Evaluation.” *JMIR MHealth and UHealth* 3, no. 3 (2015): e92. doi:10.2196/mhealth.4300.
108. Marsaux, Cyril F M, Carlos Celis-Morales, Rosalind Fallaize, Anna L Macready, Silvia Kolossa, Clara Woolhead, Clare B O’Donovan, et al. “Effects of a Web-Based Personalized Intervention on Physical Activity in European Adults: A Randomized Controlled Trial.” *Journal of Medical Internet Research* 17, no. 10 (2015): e231. doi:10.2196/jmir.4660.
109. Martinez, Remberto, and Marcos Tong. “Can Mobile Health Deliver Participatory Medicine to All Citizens in Modern Society?” In *Exploring the Abyss of Inequalities - 4th International Conference on Well-Being in the Information Society*, 83–91, 2012.
110. Maruyama, Chizuko, Mika Kimura, Hisashi Okumura, Kenji Hayashi, and Takashi Arao. “Effect of a Worksite-Based Intervention Program on Metabolic Parameters in Middle-Aged Male White-Collar Workers: A Randomized Controlled Trial.” *Preventive Medicine* 51 (2010): 11–17. doi:10.1016/j.ypmed.2010.04.008.
111. Massoudi, Barbara L, Murrey G Olmsted, Yuying Zhang, Ruth Ann Carpenter, Carolyn E Barlow, and Rachel Huber. “A Web-Based Intervention to Support Increased Physical Activity among at-Risk Adults.” *Journal Of Biomedical Informatics* 43, no. 5 Suppl (October 2010): S41-5. doi:10.1016/j.jbi.2010.07.012.
112. Mattila, Elina, Raimo Lappalainen, Juha Pärkkä, Jukka Salminen, and Ilkka Korhonen. “Use of a Mobile Phone Diary for Observing Weight Management and Related Behaviours.” *Journal of Telemedicine and Telecare* 16, no. 5 (2010): 260–64. doi:10.1258/jtt.2009.091103.
113. McMahon, Siobhan K, Beth Lewis, Michael Oakes, Weihua Guan, Jean F Wyman, and Alexander J Rothman. “Older Adults’ Experiences Using a Commercially Available Monitor to Self-Track Their Physical Activity.” *JMIR MHealth and UHealth* 4, no. 2 (2016): e35. doi:10.2196/mhealth.5120.
114. McMahon, Siobhan, Mithra Vankipuram, Eric B. Hekler, and Julie Fleury. “Design and Evaluation of Theory-Informed Technology to Augment a Wellness Motivation Intervention.” *Translational Behavioral Medicine* 4, no. 1 (2014): 95–107. doi:10.1007/s13142-013-0221-4.
115. Meng, Jingbo, Wei Peng, Soo Yun Shin, and Minwoong Chung. “Online Self-Tracking Groups to Increase Fruit and Vegetable Intake: A Small-Scale Study on

Mechanisms of Group Effect on Behavior Change." *Journal of Medical Internet Research* 19, no. 3 (2017): e63. doi:10.2196/jmir.6537.
116. Meyer, Jochen, Elke Beck, Merlin Wasmann, and Susanne Boll. "Making Sense in the Long Run: Long-Term Health Monitoring in Real Lives Jochen." In *Proceedings - 2017 IEEE International Conference on Healthcare Informatics, ICHI 2017*, 285–94, 2017. doi:10.1109/ICHI.2017.11.
117. Miyagawa, S, Y Oguma, and Y Ohgi. "Evaluation of a Community-Based Health Promotion Program with Online Off-Line Combination: The Effect of an Online Diet Record System on Self-Rated Achievement." In *Procedia Computer Science*, edited by J E Q Varajao, M M CruzCunha, R Martinho, R Rijo, N BjornAndersen, R Turner, and D Alves, 100:768–75. Procedia Computer Science. [Miyagawa, Shoko] Keio Univ, Fac Nursing & Med Care, 4411 Endo, Fujisawa, Kanagawa 2520883, Japan. [Oguma, Yuko] Keio Univ, Grad Sch Hlth Management, 4411 Endo, Fujisawa, Kanagawa 2520883, Japan. [Ohgi, Yuji] Keio Univ, Grad Sch Media & Governance, 5322 E: Elsevier Science Bv, 2016. doi:10.1016/j.procs.2016.09.223.
118. Moore, Thomas J., Nour Alsabeeh, Caroline M. Apovian, Megan C. Murphy, Gerald A. Coffman, Diana Cullum-Dugan, Mark Jenkins, and Howard Cabral. "Weight, Blood Pressure, and Dietary Benefits After 12 Months of a Web-Based Nutrition Education Program (DASH for Health): Longitudinal Observational Study." *Journal of Medical Internet Research* 10, no. 4 (2008): e52. doi:10.2196/jmir.1114.
119. Morgan, Philip J., Clare E. Collins, Ronald C. Plotnikoff, Alyce T. Cook, Bronwyn Berthon, Simon Mitchell, and Robin Callister. "Efficacy of a Workplace-Based Weight Loss Program for Overweight Male Shift Workers: The Workplace POWER (Preventing Obesity Without Eating like a Rabbit) Randomized Controlled Trial." *Preventive Medicine* 52, no. 5 (2011): 317–25. doi:10.1016/j.ypmed.2011.01.031.
120. Morgan, Philip J., David R. Lubans, Clare E. Collins, Janet M. Warren, and Robin Callister. "The SHED-IT Randomized Controlled Trial: Evaluation of an Internet-Based Weight-Loss Program for Men." *Obesity* 17, no. 11 (2009): 2025–32. doi:10.1038/oby.2009.85.
121. Morrison, Leanne G, Charlie Hargood, Sharon Xiaowen Lin, Laura Dennison, Judith Joseph, Stephanie Hughes, Danius T Michaelides, et al. "Understanding Usage of a Hybrid Website and Smartphone App for Weight Management : A Mixed-Methods Study." *Journal Of Medical Internet Research* 16, no. 10 (2014): e201. doi:10.2196/jmir.3579.
122. Morton, Katie, Stephen Sutton, Wendy Hardeman, Jacqui Troughton, Tom Yates, Simon Griffin, Melanie Davies, Kamlesh Khunti, and Helen Eborall. "A Text-Messaging and Pedometer Program to Promote Physical Activity in People at High Risk of Type 2 Diabetes: The Development of the PROPELS Follow-On Support Program." *JMIR MHealth and UHealth* 3, no. 4 (2015): e105. doi:10.2196/mhealth.5026.
123. Mummah, Sarah, Thomas N Robinson, Maya Mathur, Sarah Farzinkhou, Stephen Sutton, and Christopher D Gardner. "Effect of a Mobile App Intervention on Vegetable Consumption in Overweight Adults: A Randomized Controlled Trial." *International Journal of Behavioral Nutrition and Physical Activity* 14, no. 1 (September 2017): 125. doi:10.1186/s12966-017-0563-2.
124. Nagykaldi, Z., C. B. Aspy, A. Chou, and J. W. Mold. "Impact of a Wellness Portal on the Delivery of Patient-Centered Preventive Care." *The Journal of the*

American Board of Family Medicine 25, no. 2 (2012): 158–67. doi:10.3122/jabfm.2012.02.110130.
125. Nakajima, Kazuki, Masayuki Nambu, Tohru Kiryu, Toshiyo Tamura, and Kazuo Sasaki. “Low-Cost, Email-Based System for Self Blood Pressure Monitoring at Home.” *Journal Of Telemedicine And Telecare* 12, no. 4 (2006): 203–7. http://search.ebscohost.com/login.aspx?direct=true&db=cmedm&AN=16774703&site=ehost-live.
126. Napolitano, Melissa A, Sharon Hayes, Gary G Bennett, Allison K Ives, and Gary D Foster. “Using Facebook and Text Messaging to Deliver a Weight Loss Program to College Students.” *Obesity* 21, no. 1 (2013): 25–31. doi:10.1038/oby.2012.107.
127. Nash, Chelsea M, Katrina A Vickerman, Elizabeth S Kellogg, and Susan M Zbikowski. “Utilization of a Web-Based vs Integrated Phone/Web Cessation Program Among 140,000 Tobacco Users: An Evaluation Across 10 Free State Quitlines.” *Journal Of Medical Internet Research* 17, no. 2 (2015): e36. doi:10.2196/jmir.3658.
128. Naughton, Felix, Sarah Hopewell, Neal Lathia, Rik Schalbroeck, Chloë Brown, Cecilia Mascolo, Andy McEwen, and Stephen Sutton. “A Context-Sensing Mobile Phone App (Q Sense) for Smoking Cessation: A Mixed-Methods Study.” *JMIR MHealth and UHealth* 4, no. 3 (2016): e106. doi:10.2196/mhealth.5787.
129. Neve, Melinda, Philip J. Morgan, and Clare E. Collins. “Weight Change in a Commercial Web-Based Weight Loss Program and Its Association With Website Use: Cohort Study.” *Journal of Medical Internet Research* 13, no. 4 (2011): e83. doi:10.2196/jmir.1756.
130. O’Brien, Tara. “Mobile Health Technology Interventions to Improve the Health Status of Older Rural Women.” University of South Carolina, 2013.
131. Painter, Stefanie Lynn, Rezwan Ahmed, James O Hill, Robert F Kushner, Richard Lindquist, Scott Brunning, and Amy Margulies. “What Matters in Weight Loss? An In-Depth Analysis of Self-Monitoring.” *Journal of Medical Internet Research* 19, no. 5 (2017): e160. stefanie@retrofitme.com.
132. Park, Y J, S J Lee, N M Shin, H Shin, S Jeon, J Lee, and I Cho. “Application and Effect of Mobiletype-Bone Health Intervention in Korean Young Adult Women with Low Bone Mass: A Randomized Control Trial.” *Asian Nursing Research* 11, no. 1 (2017): 56–64. doi:10.1016/j.anr.2017.03.005.
133. Patrick, Kevin, Karen J. Calfas, Gregory J. Norman, Dori Rosenberg, Marion F. Zabinski, James F. Sallis, Cheryl L. Rock, and Lindsay W. Dillon. “Outcomes of a 12-Month Web-Based Intervention for Overweight and Obese Men.” *Annals of Behavioral Medicine* 42, no. 3 (2011): 391–401. doi:10.1007/s12160-011-9296-7.
134. Pellegrini, Christine A, Steven D Verba, Amy D Otto, Diane L Helsel, Kelliann K Davis, and John M Jakicic. “The Comparison of a Technology-Based System and an In- Person Behavioral Weight Loss Intervention.” *Obesity* 20, no. 2 (2012): 356–63. doi:10.1038/oby.2011.13.The.
135. Polzien, Kristen M, John M Jakicic, Deborah F Tate, and Amy D Otto. “The Efficacy of a Technology - Based System in a Short - Term Behavioral Weight Loss Intervention.” *Obesity* 15, no. 4 (2007): 825–30. doi:10.1038/oby.2007.584.
136. Postrach, Elisa, Rosa Aspalter, Ulf Elbelt, Michael Koller, Rita Longin, Jorg-Dieter Schulzke, and Luzia Valentini. “Determinants of Successful Weight Loss After Using a Commercial Web-Based Weight Reduction Program for Six Months: Cohort Study.” *Journal Of Medical Internet Research* 15, no. 10 (October 2013): e219. doi:10.2196/jmir.2648.

137. Ramirez, Ernesto Raul. “Discretionary Self-Monitoring of Physical Activity:A Mixed-Methods Study of Behavior Change Technique Use and Historical Physical Activity.” University of Calofornia, San Diego, 2016.
138. Redman, Leanne M, L. Anne Gilmore, Jeffrey Breaux, Diana M Thomas, Karen Elkind-Hirsch, Tiffany Stewart, Daniel S Hsia, et al. “Effectiveness of SmartMoms, a Novel EHealth Intervention for Management of Gestational Weight Gain: Randomized Controlled Pilot Trial.” *JMIR MHealth and UHealth* 5, no. 9 (2017): e133. doi:10.2196/mhealth.8228.
139. Robinson, Eric, Suzanne Higgs, Amanda J Daley, Kate Jolly, Deborah Lycett, Amanda Lewis, and Paul Aveyard. “Development and Feasibility Testing of a Smart Phone Based Attentive Eating Intervention.” *BMC Public Health* 13, no. 639 (2013): 1–7. doi:10.1186/1471-2458-13-639.
140. Robroek, Suzan J W, Suzanne Polinder, Folef J Bredt, and Alex Burdorf. “Cost-Effectiveness of a Long-Term Internet-Delivered Worksite Health Promotion Programme on Physical Activity and Nutrition: A Cluster Randomized Controlled Trial.” *Health Education Research* 27, no. 3 (June 2012): 399–410. doi:10.1093/her/cys015.
141. Ross, Kathryn M, and Rena R Wing. “Impact of Newer Self-Monitoring Technology and Brief Phone- Based Intervention on Weight Loss: A Randomized Pilot Study.” *Obesity* 24, no. 8 (2016): 1653–59. doi:10.1186/s40945-017-0033-9.Using.
142. Ryu, Hosihn, Jiyeon Jung, Jeonghyun Cho, and Dal Lae Chin. “Program Development and Effectiveness of Workplace Health Promotion Program for Preventing Metabolic Syndrome among Office Workers.” *International Journal of Environmental Research and Public Health* 14, no. 8 (August 2017): 1–14. doi:10.3390/ijerph14080878.
143. Schrager, J D, P Shayne, S Wolf, S Das, R E Patzer, M White, and S Heron. “Assessing the Influence of a Fitbit Physical Activity Monitor on the Exercise Practices of Emergency Medicine Residents: A Pilot Study.” *Jmir Mhealth and Uhealth* 5, no. 1 (2017): e2. doi:10.2196/mhealth.6239.
144. Segerståhl, Katarina, and Harri Oinas-Kukkonen. “Designing Personal Exercise Monitoring Employing Multiple Modes of Delivery: Implications from a Qualitative Study on Heart Rate Monitoring.” *International Journal Of Medical Informatics* 80, no. 12 (2011): e203–13. doi:10.1016/j.ijmedinf.2011.08.011.
145. Shapiro, Jennifer R, Tina Koro, Neal Doran, Sheri Thompson, James F Sallis, Karen Calfas, and Kevin Patrick. “Text4Diet : A Randomized Controlled Study Using Text Messaging for Weight Loss Behaviors.” *Preventive Medicine* 55, no. 5 (2012): 412–17. doi:10.1016/j.ypmed.2012.08.011.
146. Shaw, R J, D M Steinberg, J Bonnet, F Modarai, A George, T Cunningham, M Mason, et al. “Mobile Health Devices: Will Patients Actually Use Them?” *Journal of the American Medical Informatics Association* 23, no. 3 (2016): 462–66. doi:10.1093/jamia/ocv186.
147. Shuger, Sara L., Vaughn W. Barry, Xuemei Sui, Amanda McClain, Gregory A. Hand, Sara Wilcox, Rebecca A. Meriwether, James W. Hardin, and Steven N. Blair. “Electronic Feedback in a Diet- and Physical Activity-Based Lifestyle Intervention for Weight Loss: A Randomized Controlled Trial.” *International Journal of Behavioral Nutrition and Physical Activity* 8, no. 41 (2011): 1–8. doi:10.1186/1479-5868-8-41.
148. Slootmaker, Sander M., Mai J.M. Chinapaw, Albertine J. Schuit, Jacob C. Seidell, and Willem Van Mechelen. “Feasibility and Effectiveness of Online Physical

Activity Advice Based on a Personal Activity Monitor: Randomized Controlled Trial." *Journal Of Medical Internet Research* 11, no. 3 (2009): e27. doi:10.2196/jmir.1139.
149. Smith, Lisa M. "Sustained Effects of Brief Electronic Self-Monitoring as an Early Intervention for Eating Pathology." Emory University, 2008.
150. Spring, Bonnie, JM Duncan, EA Janke, Andrea T Kozak, H Gene McFadden, Andrew Demott, Alex Pictor, et al. "Integrating Technology into Standard Weight Loss Treatment: A Randomized Controlled Trial." *JAMA Internal Medicine* 173, no. 2 (2013): 105–11. doi:10.1001/jamainternmed.2013.1221.Integrating.
151. Spring, Bonnie, Kristin Schneider, H G McFadden, Jocelyn Vaughn, Andrea T Kozak, Malaina Smith, Arien C Moller, et al. "Multiple Behavior Change in Diet and Activity: A Randomized Controlled Trial Using Mobile Technology." *Archives of Internal Medicine* 172, no. 10 (2012): 789–796. doi:10.1001/archinternmed.2012.1044.Multiple.
152. Stara, Vera, Richard Harte, Mirko Di Rosa, Lorena Rossi, and Gearóid ÓLaighin. "Toward a Connected Health System for Older Adults: Lessons Learned." In *Advances in Physical Ergonomics and Human Factors*, 489:849–59, 2016. doi:10.1007/978-3-319-41694-6.
153. Stein, Natalie, Kevin Brooks, and East Street. "A Fully Automated Conversational Artificial Intelligence for Weight Loss : Longitudinal Observational Study Among Overweight and Obese Adults." *JMIR Diabetes* 2, no. 2 (2017): e28. doi:10.2196/diabetes.8590.
154. Steinberg, Dori M, Deborah F Tate, Gary G Bennett, Susan Ennett, Carmen Samuel-Hodge, and Dianne S Ward. "The Efficacy of a Daily Self-Weighing Weight Loss Intervention Using Smart Scales and Emai." *Obesity* 21, no. 9 (2013): 1789–97. doi:10.1002/oby.20396.The.
155. Sternfeld, Barbara, Sheng Fang Jiang, Teresa Picchi, Lisa Chasan-Taber, Barbara Ainsworth, and Charles P. Quesenberry. "Evaluation of a Cell Phone–Based Physical Activity Diary." *Medicine and Science in Sports and Exercise* 44, no. 3 (2012): 487–95. doi:10.1249/MSS.0b013e3182325f45.
156. Stoops, William W, Jesse Dallery, Nell M Fields, Paul A Nuzzo, E Nancy, Catherine A Martin, Baretta Casey, and Conrad J Wong. "An Internet-Based Abstinence Reinforcement Smoking Cessation Intervention in Rural Smokers." *Drug and Alcohol Dependence* 105, no. 1–2 (2009): 56–62. doi:10.1016/j.drugalcdep.2009.06.010.An.
157. Stuckey, Melanie, Elizabeth Russell-Minda, Emily Read, Claudio Munoz, Kevin Shoemaker, Peter Kleinstiver, and Robert Petrella. "Diabetes and Technology for Increased Activity (DaTA) Study: Results of a Remote Monitoring Intervention for Prevention of Metabolic Syndrome." *Journal of Diabetes Science and Technology* 5, no. 4 (July 2011): 928–35. doi:10.1177/193229681100500416.
158. Stukenberg, Elizabeth. "A Quantitative Pilot Study on the Use of a Fitness Tracker in the Preventative Management of Employees at Risk of Chronic Disease in a Health Care Facility." *Online Journal of Nursing Informatics* 19, no. 3 (2015).
159. Svetkey, LP, BC Batch, P-H Lin, SS Intille, L Corsino, CC Tyson, HB Bosworth, et al. "Cell Phone Intervention for You (CITY): A Randomized, Controlled Trial of Behavioral Weight Loss Intervention for Young Adults Using Mobile Technology." *Obesity* 23, no. 11 (2015): 2133–2141. doi:10.1007/s10549-015-3663-1.Progestin.
160. Tate, DF, EH Jackvony, and RR Wing. "A Randomized Trial Comparing Human E-Mail Counseling, Computer-Automated Tailored Counseling, and No

Counseling in an Internet Weight Loss Program." *Archives of Internal Medicine* 166, no. 15 (2006): 1620–25. doi:10.1001/archinte.166.15.1620.
161. Tate, DF, EH Jackvony, and RR Wing. "Effects of Internet Behavioral Counseling on Weight Loss in Adults at Risk for Type 2 Diabetes." *JAMA - Journal of the American Medical Association* 289, no. 14 (2003): 1833–36. doi:10.1001/jama.289.14.1833.
162. Tay, Ilona, Suzanne Garland, Alexandra Gorelik, and John Dennis Wark. "Development and Testing of a Mobile Phone App for Self-Monitoring of Calcium Intake in Young Women." *JMIR MHealth and UHealth* 5, no. 3 (2017): e27. doi:10.2196/mhealth.5717.
163. Thomas, Graham, Tricia M Leahey, and Rena R Wing. "An Automated Internet Behavioral Weight-Loss Program by Physician Referral: A Randomized Controlled Trial." *Diabetes Care* 38 (2015): 9–15. doi:10.2337/dc14-1474.
164. Thompson-felty, Claudia. "IPhone Applications and Improvement in Weight and Health Parameters: A Randomized Controlled Trial." Arizona State University, 2014.
165. Thompson, Warren G, Carol L Kuhle, Gabriel A Koepp, Shelly K Mccrady-spitzer, and A Levine. """ Go4Life "' Exercise Counseling , Accelerometer Feedback , and Activity Levels in Older People §." *Archives of Gerontology and Geriatrics* 58, no. 3 (2014): 314–19. doi:10.1016/j.archger.2014.01.004.
166. Thorndike, Anne N, Sarah Mills, Lillian Sonnenberg, Deepak Palakshappa, Tian Gao, Cindy T Pau, and Susan Regan. "Activity Monitor Intervention to Promote Physical Activity of Physicians-In-Training : Randomized Controlled Trial." *Plos One* 9, no. 6 (2014): e100251. doi:10.1371/journal.pone.0100251.
167. Thorndike, Anne N, Lillian Sonnenberg, Erica Healey, Khinlei Myint-U, Joseph C Kvedar, and Susan Regan. "Prevention of Weight Gain Following a Worksite Nutrition and Exercise Program: Randomized Controlled Trial." *American Journal of Preventive Medicine* 43, no. 1 (2012): 27–33. doi:10.1016/j.amepre.2012.02.029.Prevention.
168. Tsai, Christopher C., Gunny Lee, Fred Raab, Gregory J. Norman, Timothy Sohn, William G. Griswold, and Kevin Patrick. "Usability and Feasibility of PmEB: A Mobile Phone Application for Monitoring Real Time Caloric Balance." *Mobile Networks and Applications* 12, no. 2–3 (2007): 173–84. doi:10.1007/s11036-007-0014-4.
169. Voth, Elizabeth C, Nelly D Oelke, and Mary E Jung. "A Theory-Based Exercise App to Enhance Exercise Adherence: A Pilot Study." *JMIR MHealth and UHealth* 4, no. 2 (2016): e62. doi:10.2196/mhealth.4997.
170. Walsh, Jane C, Teresa Corbett, Michael Hogan, Jim Duggan, and Abra McNamara. "An MHealth Intervention Using a Smartphone App to Increase Walking Behavior in Young Adults: A Pilot Study." *JMIR MHealth and UHealth* 4, no. 3 (2016): e109. doi:10.2196/mhealth.5227.
171. Wang, Chih-Jau, Pimwadee Chaovalit, and Suporn Pongnumkul. "A Breastfeed-Promoting Mobile App Intervention: Usability and Usefulness Study." *JMIR Mhealth And Uhealth* 6, no. 1 (2018): e27. doi:10.2196/mhealth.8337.
172. Wang, Jing. "Social Problem Solving and Adherence to Self-Monitoring in Association with Changes in Weight and Cardiometabolic Risk Factors in a Behavioral Weight Loss Trial." University of Pittsburgh, 2010.
173. Wang, Jing, Susan M Sereika, Eileen R Chasens, Linda J Ewing, Judith T Matthews, and Lora E Burke. "Effect of Adherence to Self-Monitoring of Diet and

Physical Activity on Weight Loss in a Technology-Supported Behavioral Intervention.” *Patient Preference and Adherence* 6 (2012): 221–26.

174. Wang, Julie B., Lisa A. Cadmus-Bertram, Loki Natarajan, Martha M. White, Hala Madanat, Jeanne F. Nichols, Guadalupe X. Ayala, and John P. Pierce. “Wearable Sensor/Device (Fitbit One) and SMS Text-Messaging Prompts to Increase Physical Activity in Overweight and Obese Adults: A Randomized Controlled Trial.” *Telemedicine and E-Health* 21, no. 10 (2015): 782–92. doi:10.1089/tmj.2014.0176.

175. Ware, Lisa J., Robert Hurling, Ogi Bataveljic, Bruce W. Fairley, Tina L. Hurst, Peter Murray, Kirsten L. Rennie, et al. “Rates and Determinants of Uptake and Use of an Internet Physical Activity and Weight Management Program in Office and Manufacturing Work Sites in England: Cohort Study.” *Journal of Medical Internet Research* 10, no. 4 (2008): 1–18. doi:10.2196/jmir.1108.

176. West, Delia Smith, Courtney M Monroe, Gabrielle Turner-McGrievy, Beth Sundstrom, Chelsea Larsen, Karen Magradey, Sara Wilcox, and Heather M Brandt. “A Technology-Mediated Behavioral Weight Gain Prevention Intervention for College Students: Controlled, Quasi-Experimental Study.” *Journal Of Medical Internet Research* 18, no. 6 (2016): e133. doi:10.2196/jmir.5474.

177. Wharton, Christopher M, Carol S Johnston, Barbara K Cunningham, and Danielle Sterner. “Dietary Self-Monitoring, But Not Dietary Quality, Improves With Use of Smartphone App Technology in an 8-Week Weight Loss Trial.” *Journal of Nutrition Education and Behavior* 46, no. 5 (2014): 440–44. christopher.wharton@asu.edu.

178. Wijsman, Carolien A., Rudi G J Westendorp, Evert A L M Verhagen, Michael Catt, P. Eline Slagboom, Anton J M De Craen, Karen Broekhuizen, et al. “Effects of a Web-Based Intervention on Physical Activity and Metabolism in Older Adults: Randomized Controlled Trial.” *Journal of Medical Internet Research* 15, no. 11 (2013): e233. doi:10.2196/jmir.2843.

179. Yon, Bethany A, Rachel K Johnson, Jean Harvey-Berino, Beth Casey Gold, and Alan B Howard. “Personal Digital Assistants Are Comparable to Traditional Diaries for Dietary Self-Monitoring During a Weight Loss Program.” *Journal of Behavioral Medicine* 30, no. 2 (2007): 165–75. Rachel.johnson@uvm.edu.

180. Youm, S, and S Liu. “Development Healthcare PC and Multimedia Software for Improvement of Health Status and Exercise Habits.” *Multimedia Tools and Applications* 76, no. 17 (2017): 17751–63. doi:10.1007/s11042-015-2998-2.

181. Yu, Yang, Jingjing Li, and Jing Liu. “M-HELP: A Miniaturized Total Health Examination System Launched on a Mobile Phone Platform.” *Telemedicine and E-Health* 19, no. 11 (2013): 857–65. doi:10.1089/tmj.2013.0031.

182. Yu, Yingxiang, Yiran Lv, Bin Yao, Liguang Duan, Xiaoyuan Zhang, Lan Xie, and Cuiqing Chang. “A Novel Prescription Pedometer-Assisted Walking Intervention and Weight Management for Chinese Occupational Population.” *PloS One* 13, no. 1 (2018): e0190848. doi:10.1371/journal.pone.0190848.

183. Zhu, Haining, Joanna Colgan, Madhu Reddy, and Eun Kyoung Choe. “Sharing Patient-Generated Data in Clinical Practices: An Interview Study Abstract.” In *AMIA Annual Symposium Proceedings - American Medical Informatics Association*, 1303–12, 2016. doi:10.1177/1932296813511727.

Characteristics of Included Studies

Characteristic	Number of studies (n)	Percentages
**Publication Type**		
Scientific journal articles	n=162/183	89%
conference proceeding collections	n=13/183	7%
published theses	n=8/183	4%
**Year of Publication**		
2003	n=2/183	1%
2004	n=2/183	1%
2005	n=1/183	1%
2006	n=6/183	3%
2007	n=5/183	3%
2008	n=4/183	2%
2009	n=7/183	4%
2010	n=9/183	5%
2011	n=10/183	6%
2012	n=21/183	11%
2013	n=23/183	13%
2014	n=24/183	13%
2015	n=21/183	11%
2016	n=24/183	13%
2017	n=21/183	11%
2018	n=3/183	2%
**Location of Publication**		
North America	n=107/183	58
Europe	n=38/183	21
Australia and New Zealand	n=18/183	10
Asia	n=13/183	7
Middle East	n=1/183	1
Multiple Countries	n=6/183	3
**Study Design**		
Randomized and controlled	n=93/183	51
Quantitative and non-randomized	n=47/183	26
Mixed-methods	n=30/183	16
Qualitative	n=13/183	7
