# Supplementary material for: Electronic Patient-Generated Health Data to Facilitate Disease Prevention and Health Promotion: Scoping Review
Source: J Med Internet Res. 2019 Oct 14;21(10):e13320. doi: 10.2196/13320 (PMC6914107; doi:10.2196/13320)
Supplement: Multimedia Appendix 7 [file jmir_v21i10e13320_app7.pdf]

Multimedia Appendix: Participant characteristics

Participant Numbers

(1) Total Number of Participants across Studies: 961.512
(2) Range
    - Lowest Sample Size: 6
    - Largest Sample Size: 455341

Participant Health	Number of Studies
Publications including healthy, predominantly risk-free samples	6
Publications including high-risk samples	108
Publications including a mix of healthy and high-risk sample	30
Unclear or NA:	39

Participant Age	Number of Studies
Publications including adults across age groups	155
Publications only including participants up to 30 years	17
Publications only including participants above 50 years	11

Participant Gender	Number of Studies
Mixed	148
Males only	11
Females only	24
