# Supplementary material for: Electronic Patient-Generated Health Data to Facilitate Disease Prevention and Health Promotion: Scoping Review
Source: J Med Internet Res. 2019 Oct 14;21(10):e13320. doi: 10.2196/13320 (PMC6914107; doi:10.2196/13320)
Supplement: Multimedia Appendix 9 [file jmir_v21i10e13320_app9.pdf]

Multimedia Appendix: PGHD tools and their functionalities

Identified PGHD hardware types and associated devices

Hardware	Devices
1. non-health related products that are mostly well-integrated into daily living (n= 157/183, 86%)	Laptop, PC, tablet, mobile phone, personal digital assistant, web cam
2. health-related devices that are less societally penetrated (n=90/183, 49%)	Heart rate monitor, pedometer, physical activity trackers, Bluetooth-enabled scale, smart clothing, Carbon Monoxide meter, sleep monitor, energy monitor
3. medical devices (n=15/183, 8%)	Glucometer, digital blood pressure monitor

Most commonly addressed functionalities of electronic PGHD-Tools

PGHD-Tool functions	Number of studies reporting functionality
Enable manual PGHD collection	n= 143
Providing additional intervention components	n= 138
PGHD analysis and visualization	n= 118
Provide feedback	n= 107
Enable data transfer and sharing	n= 94
Automatic PGHD recording	n= 89
Store PGHD	n= 78
Enable interactions and social support	n= 67
